# PTEN deficiency reprogrammes human neural stem cells towards a glioblastoma stem cell-like phenotype

**DOI:** 10.1038/ncomms10068

**Published:** 2015-12-03

**Authors:** Shunlei Duan, Guohong Yuan, Xiaomeng Liu, Ruotong Ren, Jingyi Li, Weizhou Zhang, Jun Wu, Xiuling Xu, Lina Fu, Ying Li, Jiping Yang, Weiqi Zhang, Ruijun Bai, Fei Yi, Keiichiro Suzuki, Hua Gao, Concepcion Rodriguez Esteban, Chuanbao Zhang, Juan Carlos Izpisua Belmonte, Zhiguo Chen, Xiaomin Wang, Tao Jiang, Jing Qu, Fuchou Tang, Guang-Hui Liu

**Affiliations:** 1 National Laboratory of Biomacromolecules, Institute of Biophysics, Chinese Academy of Sciences, Beijing 100101, China; 2 Biodynamic Optical Imaging Center, College of Life Sciences, Peking University, Beijing 100871, China; 3 FSU-CAS Innovation Institute, Foshan 528000, China; 4 State Key Laboratory of Stem Cell and Reproductive Biology, Institute of Zoology, Chinese Academy of Sciences, Beijing 100101, China; 5 Department of Pathology, Carver College of Medicine, University of Iowa, Iowa City, Iowa 52242, USA; 6 Gene Expression Laboratory, Salk Institute for Biological Studies, 10010 North Torrey Pines Road, La Jolla, California 92037, USA; 7 Department of Molecular and Cellular Physiology, Stanford University School of Medicine, Stanford, California 94305, USA; 8 Universidad Católica San Antonio de Murcia (UCAM) Campus de los Jerónimos, No 135 Guadalupe, 30107 Murcia, Spain; 9 Research Center for Translational Medicine, Shanghai East Hospital, School of Life Sciences and Technology, Tongji University, Shanghai 200092, China; 10 Beijing Institute for Brain Disorders, Beijing 100069, China; 11 Cell Therapy Center, Xuanwu Hospital Capital Medical University, Beijing 100053, China; 12 Ministry of Education Key Laboratory of Cell Proliferation and Differentiation, Beijing 100871, China; 13 Center for Molecular and Translational Medicine, CMTM, Beijing 100101, China; 14 Peking-Tsinghua Center for Life Sciences, Peking University, Beijing 100871, China

## Abstract

PTEN is a tumour suppressor frequently mutated in many types of cancers. Here we show that targeted disruption of PTEN leads to neoplastic transformation of human neural stem cells (NSCs), but not mesenchymal stem cells. PTEN-deficient NSCs display neoplasm-associated metabolic and gene expression profiles and generate intracranial tumours in immunodeficient mice. PTEN is localized to the nucleus in NSCs, binds to the *PAX7* promoter through association with cAMP responsive element binding protein 1 (CREB)/CREB binding protein (CBP) and inhibits *PAX7* transcription. PTEN deficiency leads to the upregulation of PAX7, which in turn promotes oncogenic transformation of NSCs and instates ‘aggressiveness' in human glioblastoma stem cells. In a large clinical database, we find increased PAX7 levels in PTEN-deficient glioblastoma. Furthermore, we identify that mitomycin C selectively triggers apoptosis in NSCs with PTEN deficiency. Together, we uncover a potential mechanism of how PTEN safeguards NSCs, and establish a cellular platform to identify factors involved in NSC transformation, potentially permitting personalized treatment of glioblastoma.

## Abstract (web summary)

The tumor suppressor *PTEN* is often mutated or lost in glioblastoma. Here, the authors demonstrate that in neuronal stem cells PTEN trans-represses *PAX7* gene expression and PTEN deficiency promotes PAX7-dependent neoplastic transformation.

## Graphical abstract

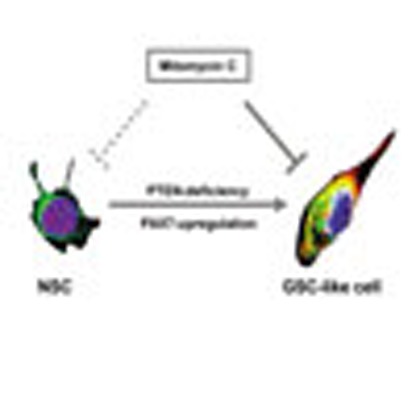

Phosphatase and tensin homolog (PTEN) is a potent tumour suppressor whose loss-of-function mutations are often encountered in human cancers. *PTEN* mutations are observed in 60% of glioblastoma multiforme (GBM) and are among the most frequent genetic alterations linked to GBM^1^. GBMs bearing *PTEN* loss-of-function mutations are usually associated with increased invasive behaviours and drug resistance^2^,^3^,^4^. Glioblastoma stem cells (GSCs), the tumorigenic component of GBM, represent a rare cell population that are resistant to conventional radio- or chemo-therapy, and are presumably involved in cancer relapse^5^,^6^. Evidence from mouse tumour models reveals neural precursor/stem cells as the cell-of-origins for GBM or GSCs^7^,^8^,^9^, and GBM is postulated to be derived from transformed neural stem cells (NSCs) that undergo carcinogenic hits^10^. The high mutation rate of *PTEN* in GBM suggests its potential as one of the initiating oncogenic events or a key factor in promoting cancer aggressiveness, similarly as seen in endometrial cancer^11^. The correlation between PTEN deficiency and poor prognosis suggests a more complex role of PTEN loss in GBM progression. These observations raise an interesting question, that is, how PTEN loss leads to GBM initiation or promotes its progression?

Mouse models have been successfully used to investigate the roles of genetic mutations in triggering oncogenic NSC transformation and/or mediating GBM pathogenesis^12^,^13^. The known differences between mouse and human cancer biology, including differential telomere length, distinct utilization of p16^INK4a^-RB versus p53 signalling and different sensitivity to anti-tumour drugs, however, have limited the degree to which insights derived from mouse models can be directly translated to human applications^14^,^15^,^16^. The advances in human stem cells and targeted gene editing technology have opened a new avenue for disease modelling and drug discovery^17^. Although many genetic disease models that are linked to development and ageing have been developed using human embryonic stem cells (ESCs) or induced pluripotent stem cells (iPSCs)^17^,^18^,^19^,^20^,^21^,^22^,^23^,^24^, very few human cancer models employing targeted genetic mutations in adult stem cells have been established for gaining mechanistic insights or testing drug efficacies^25^,^26^.

Considering the potential of NSCs being the cell-of-origin for human GBM, and PTEN deletion has frequently been reported in GBM, we hypothesize that PTEN functions as a gatekeeper to protect human NSCs from neoplastic transformation. Accordingly, we generated PTEN-deficient human NSCs by targeted gene editing. PTEN deficiency resulted in a reprogramming of NSCs towards a GSC-like phenotype in a highly lineage-specific mechanism primarily through transcriptional activation of *PAX7*, a pathway that is also linked to the aggressive characteristics of GSCs in GBM patients.

## Results

### PTEN deficiency leads to neoplastic features in NSCs

**Figure 1:**
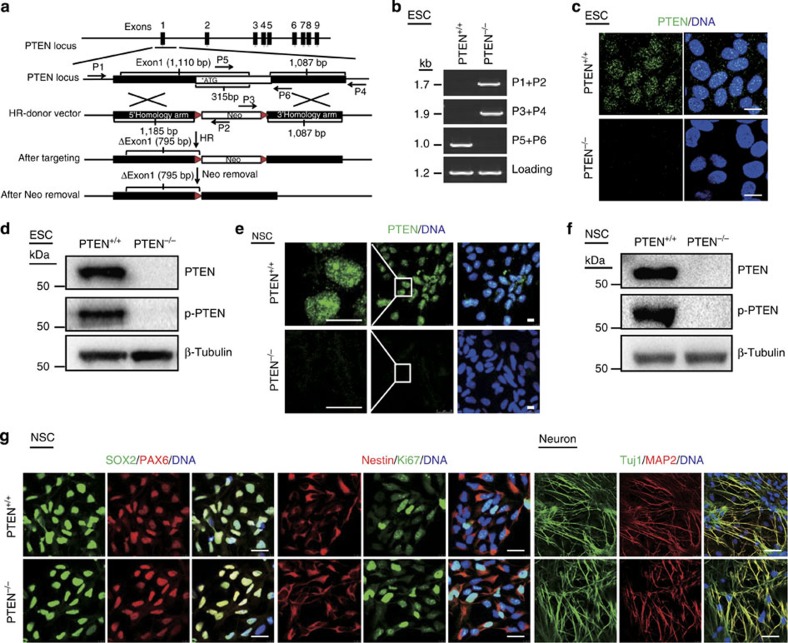
Generation and characterization of PTEN-deficient NSCs. (**a**) Schematic representation of TALEN-based *PTEN* targeting strategy. Primers used for **b** are shown as arrows (P1–P6). The donor vector includes a neomycin-resistance cassette (Neo) allowing for positive selection. (**b**) PCR analysis of WT and *PTEN*^−/−^ ESCs using primer pairs indicated (P1+P2: 1,744 bp; P3+P4: 1,872 bp; P5+P6: 1,035 bp). (**c**) Immunofluorescence analysis performed on WT and *PTEN*^−/−^ ESCs with an anti-PTEN antibody. PTEN was absent in the *PTEN*^−/−^ ESCs. Nuclei were stained with Hoechst 33342. Scale bars, 12.5 μm. (**d**) Immunoblotting verified the absence of PTEN protein in *PTEN*^−/−^ ESCs with anti-PTEN and anti-phospho-PTEN (Ser380) antibodies. β-Tubulin was used as loading control. (**e**) Immunostaining of PTEN in WT and *PTEN*^−/−^ NSCs with an anti-PTEN antibody. Scale bars, 10 μm. (**f**) Immunoblotting analysis of PTEN and phospho-PTEN in WT and *PTEN*^−/−^ NSCs. (**g**) Immunostaining of neural progenitor- (left) and neuron- (right) specific markers in WT and *PTEN*^−/−^ NSCs (left) and their neuronal derivatives (right). Scale bars, 25 μm (NSC) and 50 μm (neuron).

To generate isogenic PTEN-null human NSC lines, we first utilized transcription activator-like effector nuclease (TALEN)-mediated homologous recombination (HR) to delete the exon 1 of *PTEN* gene in human ESCs (Fig. 1a). Successful gene targeting at *PTEN* locus was verified by genomic PCR (Fig. 1b). Immunofluorescence staining revealed a punctate staining pattern of PTEN in the nucleus of wild-type (WT) ESCs, which was absent in *PTEN* homozygous knockout (*PTEN*^−/−^) ESCs (Fig. 1c). Western blot further confirmed the loss of PTEN protein in *PTEN*^−/−^ ESCs (Fig. 1d). The deficiency of PTEN, however, did not alter ESC-associated features such as morphology, DNA hypo-methylation at *OCT4* promoter and the expression of pluripotency markers OCT4, SOX2, NANOG and TRA-1-81 (Supplementary Fig. 1a–c). *PTEN*^−/−^ ESCs also maintained the capability of differentiating into endoderm, mesoderm and ectoderm lineages *in vivo*, as did WT ESCs (Supplementary Fig. 1d).

To study the role of PTEN in NSCs, we derived NSCs from both WT and *PTEN*^−/−^ ESCs. We confirmed the loss of PTEN in *PTEN*^−/−^ NSCs (Fig. 1e,f and Supplementary Fig. 2a). Both the WT and *PTEN*^−/−^ NSCs exhibited typical features of neural progenitors, including the expression of neural progenitor markers PAX6, SOX2 and Nestin, the absence of pluripotency-related antigen TRA-1-81, the hyper-methylation of *OCT4* promoter, as well as the enrichment of H3K4me3 levels at *PAX6* and *Nestin* loci (Fig. 1g and Supplementary Fig. 2b–f). More importantly both WT and *PTEN*^−/−^ NSCs were able to efficiently differentiate into neurons and astrocytes *in vitro* (Fig. 1g and Supplementary Fig. 2g), confirming their NSC identity.

**Figure 2:**
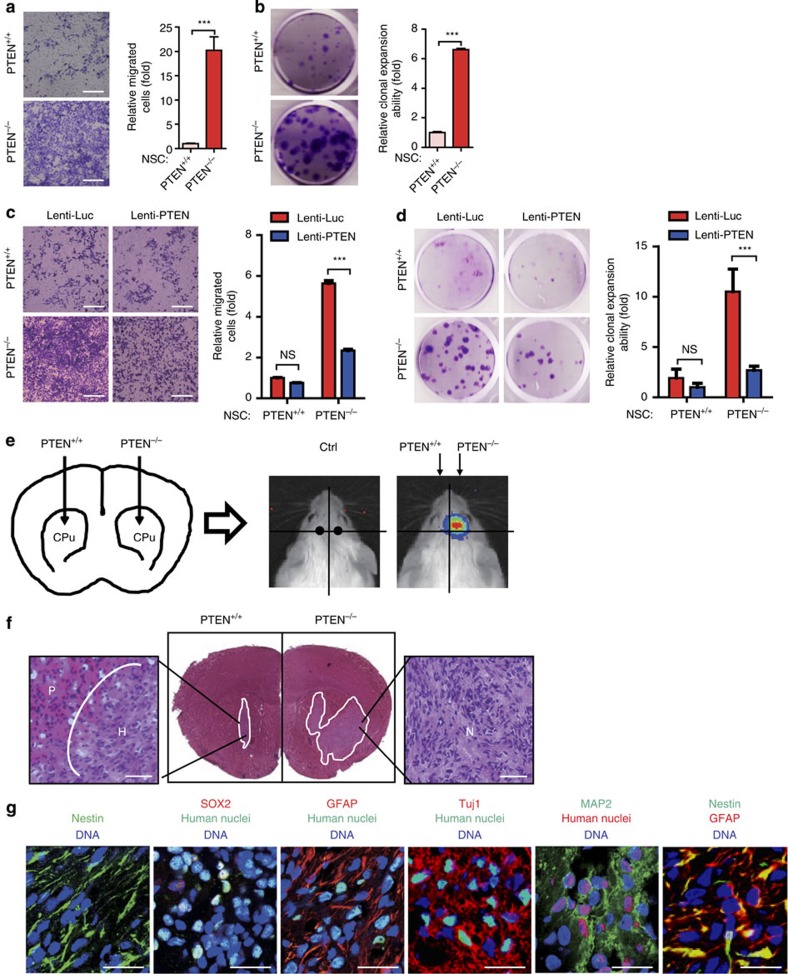
PTEN-deficient NSCs demonstrated neoplastic features *in vitro* and *in vivo*. (**a**) Migration abilities of WT and *PTEN*^−/−^ NSCs were evaluated by Transwell assays. Relative cell migration efficiency was determined. Data are shown as mean±s.e.m. *n*=3. ***P<0.001 (*t*-test). Scale bars, 1 mm. (**b**) Clonal expansion analysis in WT and *PTEN*^−/−^ NSCs. Crystal violet staining-positive cells were calculated and presented as fold induction using Image J. Data are shown as mean±s.e.m. *n*=4. ***P<0.001 (*t*-test). (**c**) Cell migration analyses of NSCs transduced with a PTEN expression vector (Lenti-PTEN) or a control vector (Lenti-Luc). Data are shown as mean±s.e.m. *n*=6. ***P<0.001 (*t*-test); NS, not significant. Scale bars, 1 mm. (**d**) Clonal expansion analysis of NSCs transduced with Lenti-PTEN or Lenti-Luc. Data are shown as mean±s.e.m. *n*=3. ***P<0.001 (*t*-test); NS, not significant. (**e**) Representative photon flux images from the brain of NOD/SCID mice implanted with WT (left) and *PTEN*^−/−^ (right) NSCs expressing luciferase (also see Supplementary Fig.6a for details). Images were taken 20 min after L-luciferin was injected intraperitoneally (i.p.). *n*=3. (**f**) H&E staining and photomicrograph of a section of entire brain showing dramatic neoplastic expansion of *PTEN*^−/−^ NSCs relative to WT control (2 × 10^6^ cells per injection), 35 days after cells were injected into the contralateral corpus striatum. H represents residual human NSC graft without expansion, N represents neoplasm and P represents mouse brain parenchyma. Scale bars, 500 μm. (**g**) Immunostaining of brain sections described in **f** with antibodies against the indicated antigens. Human nuclei, human nucleus antigen. DNA was stained with Hoechst 33342. Scale bars, 25 μm.

Next, we investigated whether PTEN-deficient NSCs could have acquired neoplastic potentials. *PTEN*^−/−^ NSCs exhibited accelerated growth under suspended neurosphere culturing condition (Supplementary Fig. 3a), accompanied by a mild increase in the population of cells retained in the S and G2/M phases and a concomitant decrease in the population of cells in the G0/G1 phases (Supplementary Fig. 3b). Consistent with an acquired growth advantage, *PTEN*^−/−^ NSCs exhibited enlarged nuclei size, increased number of nucleoli and elevated rRNA transcripts (Supplementary Fig. 3c–e). It has been reported that PTEN-loss is associated with genomic instability in mouse embryonic fibroblasts (MEFs)^27^. We performed karyotyping and genome-wide copy number variation (CNV) analyses by deep sequencing and did not observe any discernible chromosomal aberrations in *PTEN*^−/−^ NSCs compared with the WT NSCs (Supplementary Fig. 3f, g). In agreement with a neoplastic potential, *PTEN*^−/−^ NSCs showed resistance to stresses-induced cell death (Supplementary Fig. 3h,i)^28^,^29^, as well as significant elevation in cell migration and clonal expansion abilities (Fig. 2a,b and Supplementary Fig. 4a,b). Re-introduction of PTEN into the *PTEN*^−/−^ NSCs partially rescued these neoplastic phenotypes (Fig. 2c,d and Supplementary Fig. 4c,d). In addition, knockdown of *PTEN* using small hairpin (sh) RNA in either ESC- or iPSC-derived NSCs recapitulated the aggressive phenotypes observed in *PTEN*^−/−^ NSCs (Supplementary Figs 4e,f and 5a–e). Altogether, these *in vitro* experiments demonstrated that PTEN deficiency endowed NSCs with neoplastic potential.

Next we explored whether PTEN deficiency is linked to tumour initiation in NSCs using an *in vivo* mouse model. We implanted WT or PTEN-deficient NSCs expressing luciferase into the brains of immunocompromised NOD/SCID mice. PTEN-deficient NSCs were able to grow efficiently and form intracranial tumours *in vivo* as evidenced by both positive luminescence and magnetic resonance imaging (MRI) signals (Fig. 2e and Supplementary Fig. 6a–c). Similar to primary human NSCs^30^, WT ESC-derived NSCs did not expand in the brain (Fig. 2e). Haematoxylin and eosin (H&E) staining of brain slices showed a substantial expansion of the grafted *PTEN*^−/−^ NSCs, with characteristics of neoplastic cells intruding adjacent normal brain tissue and histological resemblance of intracranial tumour derived from the grafted human GSCs (Fig. 2f and Supplementary Fig. 6e)^30^,^31^,^32^,^33^. Occasionally we observed invasion of neoplastic cells into other brain regions, intra-tumour vasculatures and non-classic necrosis in the mouse brain implanted with *PTEN*^−/−^ NSCs for a longer course (∼70 days; Supplementary Fig. 6e). *PTEN*^−/−^ NSC-derived neoplasm also expressed typical mixed markers of GBM including Nestin, SRY box 2 (SOX2), glial fibrillary acidic protein (GFAP), neuron-specific beta -III tubulin (Tuj1) and microtubule-associated protein 2 (MAP2) (Fig. 2g)^33^,^34^. In agreement with several studies using GSCs^35^,^36^, the neoplastic phenotype in *PTEN*^−/−^ NSCs was mediated by their CD133-positive, rather than CD133-negative population (Supplementary Fig. 6f). The *in vivo* aggressiveness of *PTEN*^−/−^ NSCs was able to be suppressed by expressing exogenous *PTEN* (Supplementary Fig. 6d), suggesting a direct involvement of PTEN in suppressing the neoplastic phenotype in NSCs.

A full-fledged cancer phenotype normally needs a combination of multiple oncogenic mutations. To test whether the neoplastic phenotype in PTEN-deficient NSCs can be aggravated by inactivation of another tumour suppressor, we knocked down one of the most well-known tumour suppressor p53 in PTEN-deficient NSCs^37^. As expected, we observed dramatic neoplastic transformation in NSCs when p53 and PTEN were simultaneously inactivated (Supplementary Fig. 7a–e). Next, we examined whether the intracranial niche of the brain was required for tumorigenesis mediated by PTEN deletion in NSCs. When *PTEN*^−/−^ NSCs were implanted into NOD/SCID mice via the subcutaneous route, we did not detect any neoplasm-like growth, suggesting that *PTEN*^−/−^ NSCs required a supportive brain environment for tumour initiation.

We also investigated whether PTEN deletion had impact on neoplastic transformation in other human adult stem cells. To this end, we differentiated *PTEN*^−/−^ ESCs to mesenchymal stem cells (MSCs)^22^,^23^. The *PTEN*^−/−^ MSCs expressed specific markers, such as CD73, CD90, CD105, and were absent of MSC-irrelevant markers, including CD34, CD43 and CD45 (Supplementary Fig. 8a,b). The *PTEN*^−/−^ MSCs exhibited typical features of premature senescence, including the positive SA-β-Gal staining, expression of senescence-related gene transcripts and the compromised migration and clonal expansion (Supplementary Fig. 8c–g). Likewise, knockdown of PTEN using shRNA led to senescence in WT human MSCs (Supplementary Fig. 8h,i). Similar accelerated ageing phenotypes were also observed in the fibroblast-like cells derived from *in vivo* differentiated *PTEN*^−/−^ ESCs (Supplementary Fig. 8j). The pro-senescence effect observed in MSCs appears to be specific to PTEN deficiency, as knockdown of p53 in MSCs led to strengthened proliferation and migration instead (Supplementary Figs 7e and 8k,l). Herein, these observations indicate that PTEN deficiency preferentially induces neoplastic transformation in NSCs by promoting proliferation and invasiveness; however, in the case of mesodermal cell types such as MSCs and fibroblasts, PTEN deletion primarily triggers cellular senescence.

### *PTEN* ^−/−^ NSCs display oncogenic gene expression changes

As PTEN is a suppressor of PI3K-AKT pathway primarily via its activity as a lipid phosphatase^38^,^39^, we next examined the downstream signalling pathways that are regulated by PTEN. *PTEN*^−/−^ NSCs exhibited an activated AKT pathway as evidenced by the phosphorylation of AKT and its downstream targets such as mammalian target of rapamycin (mTOR) and glycogen synthase kinase 3 beta (GSK3β) when compared with the WT NSCs (Supplementary Fig. 9a,b). In contrast, AKT pathway activities were not affected by PTEN status in MSCs (Supplementary Fig. 9a), suggesting a different regulatory mechanism for AKT activation in MSCs.

**Figure 3:**
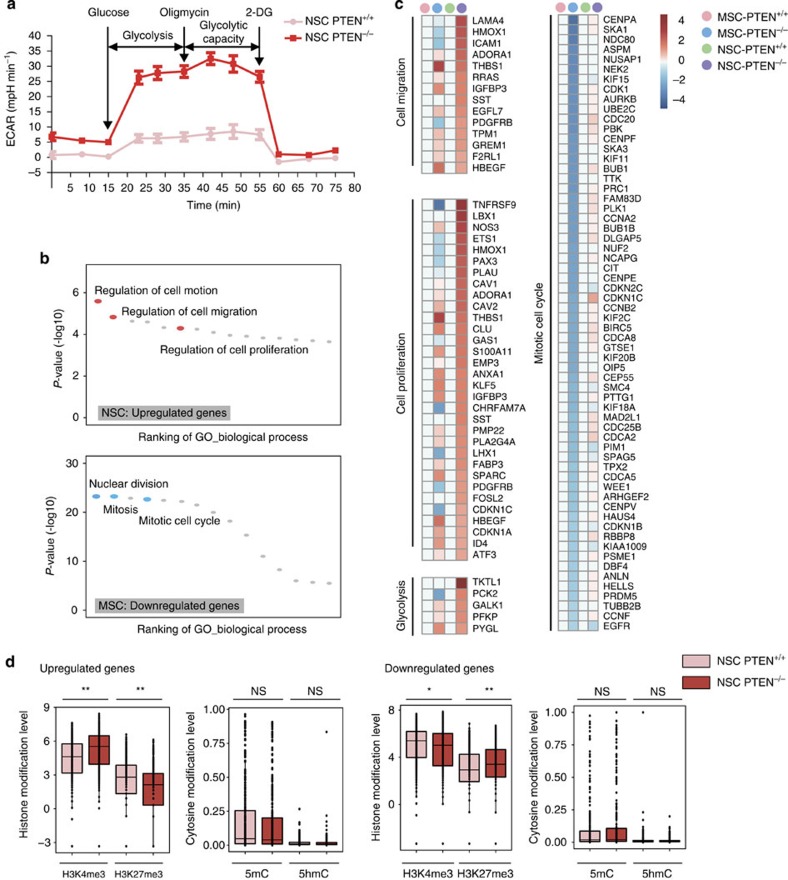
Transcriptome and epigenome analyses in PTEN-deficient stem cells. (**a**) Glycolysis flux analysis measured by extracellular acidification rates (ECARs) in PTEN^+/+^ and PTEN^−/−^ NSCs. *n*=5. (**b**) GO biological process analysis of genes upregulated (*q*-value<0.05, FC[PTEN^−/−^/PTEN^+/+^]>2) after PTEN knockout in NSCs and genes downregulated (*q*-value<0.05, FC[PTEN^−/−^/PTEN^+/+^]<0.5) after PTEN knockout in MSCs. (**c**) Heat map displaying the upregulated genes (*q*-value<0.05, FC[PTEN^−/−^/PTEN^+/+^]>2) in PTEN-deficient NSCs (left) and the downregulated genes (q-value <0.05, FC[PTEN^−/−^/PTEN^+/+^]<0.5) in PTEN-deficient MSCs (right). The representative enriched GO terms of biological processes were shown at the left side of each panel. (**d**) Boxplots showing H3K4me3, H3K27me3, 5mC and 5hmC levels in the region of 1 kb upstream and 1 kb downstream of TSS of differentially expressed genes between PTEN^−/−^ and WT NSCs including the upregulated genes (left panels, *q*-value<0.05, FC[PTEN^−/−^/PTEN^+/+^]>2; *n*=326) and downregulated genes (right panels, *q*-value <0.05, FC[PTEN^−/−^/PTEN^+/+^]<0.5; *n*=281). *P value <0.05 (Wilcoxon signed-rank test); **P value <0.01 (Wilcoxon signed-rank test); NS, not significant.

Given that the AKT and mTOR activations are important for metabolic regulation of cancer cells, we further investigated the metabolic changes conferred by PTEN deficiency in NSCs. Mass spectrometry-based screening identified 16 metabolites were significantly upregulated in *PTEN*^−/−^ NSCs compared with WT NSCs (Supplementary Fig. 9c,d and Supplementary Data 1). Among the 16 metabolites some belong to glycolytic pathway including glucose, glucose-6-phosphate and fructose-6-phosphate. Seahorse analysis indicated an increased glycolysis activity in *PTEN*^−/−^ NSCs (Fig. 3a and Supplementary Fig. 9e). Consistently, *PTEN*^−/−^ NSCs produced threefold more lactate acid content than that produced by WT NSCs (Supplementary Fig. 9f), and the ratio between ADP or AMP and ATP were increased in *PTEN*^−/−^ NSCs relative to that of WT NSCs (Supplementary Fig. 9c and Supplementary Data 1). These data strongly support that *PTEN*^−/−^ NSCs have dysregulated glucose metabolism and favour aerobic glycolysis, a feature characteristic of GBM and most cancer cells^40^,^41^. The PTEN-deficiency-induced metabolic change was specific to NSCs, because these metabolites did not increase in *PTEN*^−/−^ ESCs when compared with WT ESCs (Supplementary Figs 9d,f and Supplementary Data 1).

Next, we investigated the impact of PTEN loss on global transcriptomic landscape of NSCs. We performed RNA-seq analyses for both WT and *PTEN*^−/−^ NSCs as well as MSCs. Using a threshold of twofold change with *q*-values less than 0.05, we identified 607 genes differentially expressed between WT and *PTEN*^−/−^ NSCs, among which 326 were upregulated (Supplementary Fig. 10a–e and Supplementary Data 2). Gene ontology (GO) terms enriched in upregulated genes are linked to GSC-related functions (Fig. 3b and Supplementary Data 2). Notably, most of these genes belong to signalling pathways important for cell motion, migration and proliferation (Fig. 3b,c, Supplementary Fig. 10f,g and Supplementary Data 2). Consistent with the metabolic changes, a number of enzymes involved in glycolysis were upregulated in *PTEN*^−/−^ NSCs (Fig. 3c and Supplementary Fig. 10f,g). These gene expression changes were partially rescued by re-introduction of PTEN in *PTEN*^−/−^ NSCs (Supplementary Fig. 10h). In contrast, PTEN deletion in MSCs resulted in differential expression of 1,854 genes (Supplementary Fig. 10a–e). Consistent with the non-neoplastic phenotype in *PTEN*^−/−^ MSCs, PTEN deficiency led to a coordinated downregulation of genes required for cell cycle process (Fig. 3b,c and Supplementary Data 2), which is in agreement with a molecular signature of accelerated senescent MSCs^23^. Of note is that only few gene expression changes caused by PTEN deficiency overlap between NSCs and MSCs (Supplementary Fig. 10d and Supplementary Data 2), serving a possible explanation for the dramatically different cellular phenotypes observed between PTEN-deficient NSCs and MSCs.

To explore whether global transcriptome change in *PTEN*^−/−^ NSCs are linked to epigenetic alterations, we performed chromatin immunoprecipitation sequencing (ChIP-seq) to map genome-wide distribution of H3K4me3, H3K27me3, as well as reduced representation bisulfite sequencing (RRBS) and Tet-assisted RRBS (TA-RRBS) for 5-methylcytosine (5mC) and 5-hydroxymethylcytosine (5hmC) in WT or *PTEN*^−/−^ NSCs (Fig. 3d and Supplementary Fig. 11a,b). Notably, the upregulated genes showed significant increase of H3K4me3 and decrease of H3K27me3, whereas the downregulated genes were associated with diminished H3K4me3 and elevated H3K27me3, at their promoter regions (Fig. 3d). We did not observe any global associations between 5mC (or 5hmC) and gene expression changes (Fig. 3d). These observations suggested that changes in histone modifications including H3K4me3 and H3K27me3 could account for PTEN-loss-induced transcriptional dysregulation in *PTEN*^−/−^ NSCs.

### PAX7 mediates oncogenic features in *PTEN* ^−/−^ NSCs

Among the upregulated genes, transcription factor *PAX7* was at the top of the list and expressed 155-fold higher in *PTEN*^−/−^ NSCs than in WT NSCs (Supplementary Data 2). Also it is known that elevated levels of PAX7 in embryonal rhabdomyosarcoma cells are associated with increased migration and invasiveness^42^,^43^. Thus, we focused on a potential role of PAX7 in regulating neoplastic transformation of NSCs.

**Figure 4:**
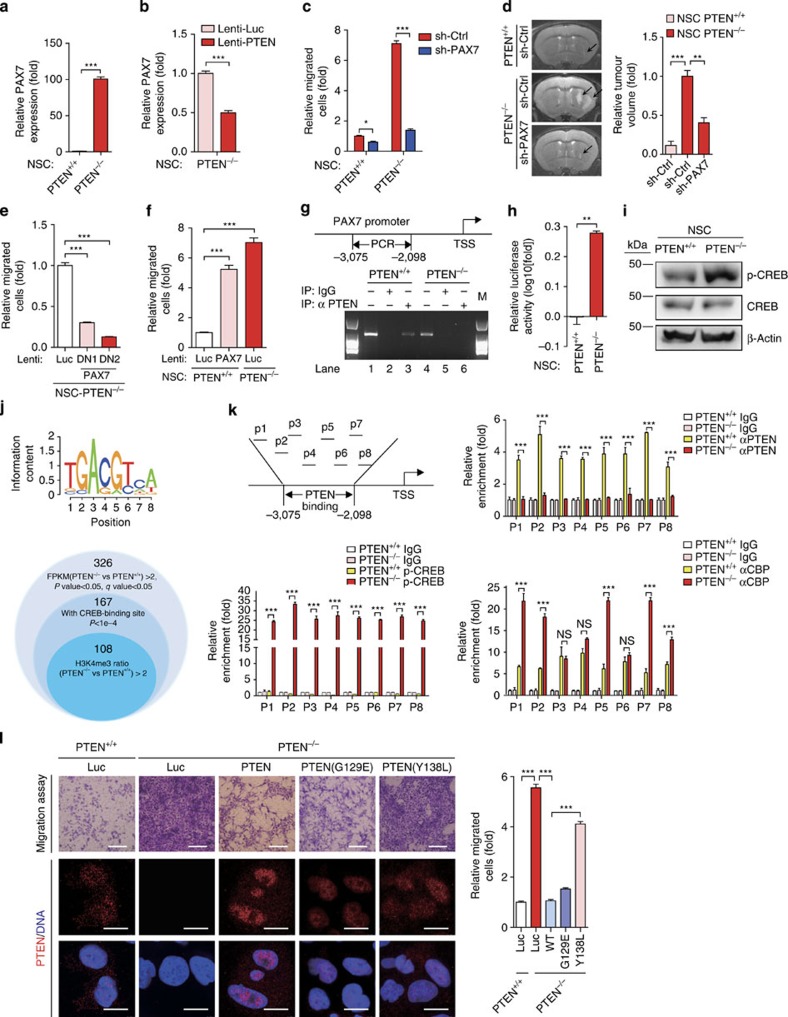
Transcriptional activation of PAX7 underlies oncogenic features in PTEN-deficient NSCs. (**a**) RT–qPCR analyses of *PAX7* expression in WT and *PTEN*^−/−^ NSCs. *n*=3. ***P<0.001 (*t*-test). (**b**) RT–qPCR showing reduced transcripts of *PAX7* in *PTEN*^−/−^ NSCs transduced with lentivirus encoding PTEN. *n*=3. ***P<0.001 (*t*-test). (**c**) Cell migration analysis in WT and *PTEN*^−/−^ NSCs transduced with control shRNA or PAX7 shRNA. *n*=5. *P<0.05 (*t*-test); and ***P<0.001 (*t*-test). (**d**) MRI analysis of intracranially implanted WT and *PTEN*^−/−^ NSCs pre-transduced with control or PAX7 shRNA. Relative volumes of tumours are presented. *n*=4. **P<0.01 (*t*-test), and ***P<0.001 (*t*-test). (**e**) Cell migration analysis in *PTEN*^−/−^ NSCs transduced with the lentiviral vector encoding dominant negative mutants of PAX7 (DN1: a.a.1–263; DN2: a.a.1–318) or a luciferase control (Luc). *n*=3. ***P<0.001 (*t*-test). (**f**) Cell migration analysis in WT NSCs transduced with the lentiviral vector encoding PAX7. *n*=3. ***P<0.001 (*t*-test). (**g**) ChIP-PCR showing the association of endogenous PTEN with *PAX7* promoter in WT NSCs. The *PAX7* promoter was amplified by PCR from either genomic DNA as input (lanes 1 and 4) or anti-PTEN immunoprecipitated DNA (lanes 3 and 6). (**h**) Luciferase reporter assay showed that the PTEN-docking DNA element described in **g** has a higher *cis*-activation ability when PTEN was depleted in NSCs. *n*=3. **P<0.01 (*t*-test). (**i**) Immunoblotting analysis of CREB and phospho-CREB (Ser133) expression in WT and *PTEN*^−/−^ NSCs. β-Actin was used as loading control. (**j**) Upper panel: Identification of CRE-containing genes in the human genome by the FIMO software tool. Lower panel: Bioinformatic analysis predicated that 167 (51.2%) of 326 genes upregulated in *PTEN*^−/−^ NSCs are potential CREB-targets. (**k**) ChIP-qPCR analysis for PTEN, p-CREB and CBP enrichment within the PTEN-docking DNA region at the *PAX7* promoter in WT and *PTEN*^−/−^ NSCs. P1–P8 indicate the different sub-regions. ***P<0.001 (*t*-test); NS, not significant. (**l**) Cell migration analysis (top panels) of *PTEN*^−/−^ NSCs transduced with the lentiviral vector encoding PTEN, PTEN(G129E), PTEN(Y138L) or a luciferase control (Luc), and the expression of PTEN was determined by immunofluorescence (lower panels). Scale bars, 1 mm (migration assay) and 50 μm (IF). *n*=3. ***P<0.001 (*t*-test).

We confirmed the elevated PAX7 expression in *PTEN*^−/−^ NSCs relative to WT NSCs, using reverse transcription–quantitative PCR (RT-qPCR) and western blotting (Fig. 4a and Supplementary Fig. 12a). In contrast, the expression of PAX7 in *PTEN*^−/−^ MSCs did not increase when compared with WT MSCs (Supplementary Fig. 12b). The upregulation of PAX7 in *PTEN*^−/−^ NSCs was directly caused by the absence of PTEN as reintroduction of PTEN in these cells repressed PAX7 expression (Fig. 4b and Supplementary Fig. 12c).

To investigate whether PAX7 mediates the neoplastic effect of PTEN deficiency during the NSC transformation, *PTEN*^−/−^ NSCs were transduced with lentiviral particles encoding two independent PAX7 shRNAs, which resulted in downregulation of PAX7 in the *PTEN*^−/−^ NSCs (Supplementary Fig. 12d). Silencing PAX7 effectively inhibited the clonal expansion and migratory capabilities of *PTEN*^−/−^ NSCs *in vitro*, as well as the intracranial neoplasm formation *in vivo* (Fig. 4c,d and Supplementary Fig. 12d–h). Ectopic expression of PAX7 dominant-negative mutants, which do not contain the transactivation domain, also inhibited the neoplastic features of *PTEN*^−/−^ NSCs (Fig. 4e), suggesting a critical role of the transcriptional activity of PAX7 in promoting neoplastic phenotypes in NSCs. To examine if elevated level of PAX7 alone is able to cause the neoplastic changes in WT NSCs, we overexpressed PAX7 in WT NSCs, to a similar level as in *PTEN*^−/−^ NSCs, and found that ectopic PAX7 expression recapitulated the major phenotypes caused by PTEN depletion, including an increase of *in vitro* cellular migration and *in vivo* neoplastic expansion (Fig. 4f and Supplementary Fig. 13a–c). In addition, overexpression of *PAX7* in WT NSCs resulted in a gene expression signature resembling that of *PTEN*^−/−^ NSCs and distinct from WT NSCs (Supplementary Fig. 13d,e and Supplementary Data 3). We found that majority of genes (19/23) whose transcripts were co-upregulated by *PAX7* overexpression and *PTEN*-knockout are associated with tumour-promoting or increased cellular mobility (Supplementary Fig. 13f and Supplementary Data 3). Therefore, PAX7 appears to be one of the key factors mediating neoplastic potential of PTEN-deficient NSCs.

Given that PTEN was predominantly localized to the nucleus in NSCs (Fig. 1e), and emerging evidence suggests that PTEN associates with chromatin DNA and is involved gene transcription regulation^27^,^44^, we thus reason that PTEN may repress *PAX7* transcription through direct binding to its promoter. A ChIP-based promoter mapping revealed a 981-bp DNA region upstream of the transcription starting site (TSS) of *PAX7* as being a docking site for PTEN in WT NSCs, but not in WT MSCs (Fig. 4g and Supplementary Fig. 14a). When cloned in a luciferase reporter, this region showed higher *cis*-activation ability when PTEN was depleted in NSCs (Fig. 4h). Bioinformatics analysis predicted several binding sites in this region for transcription factor CREB and its co-activator CBP/p300 (http://www.cbrc.jp/research/db/TFSEARCH.html). Given that CREB is a direct phosphatase substrate of nuclear PTEN^45^, we hypothesized that interplay between PTEN and CREB account for the PTEN-mediated transcriptional repression of *PAX7* as well as other genes in NSCs. Supporting this hypothesis, *PTEN*^−/−^ NSCs demonstrated elevated levels in the phosphorylated form of CREB (p-CREB; Fig. 4i). Bioinformatics predicted that 167 (51.2%) of 326 genes that were upregulated in *PTEN*^−/−^ NSCs are potentially CREB targets (*P*<10E^−4^; Fig. 4j). Among these genes, the promoters of 108 genes showed clear enrichments of the active histone mark H3K4me3, suggesting a permissive/active gene transcription state (Fig. 4j, Supplementary Fig. 14b and Supplementary Data 4). As for *PAX7* gene, ChIP-quantitative-PCR showed that in WT NSCs PTEN and CREB/CBP co-occupied within the 981-bp DNA region at the *PAX7* promoter (Fig. 4k). Co-immunoprecipitation (IP) and ChIP analyses further indicated that PTEN formed a protein complex with CREB and CBP (Supplementary Fig. 14c,d)^45^,^46^. Moreover, depletion of PTEN in WT NSCs resulted in an elevated occupation of p-CREB and/or CBP as well as H3K4me3 at the *PAX7* promoter (Fig. 4k and Supplementary Fig. 14b). Using a GAL4-based reporter assay, we found that the co-activator activity of CBP was elevated in PTEN-deficient NSCs, which could be rescued by reintroduction of PTEN (Supplementary Fig. 14e). These results support a model that PTEN deficiency led to a de-repression of CREB/CBP machinery at the *PAX7* promoter, resulting in activation of *PAX7* transcription (Supplementary Fig. 14f).

Next, we investigated whether over-activation of CREB/CBP upon PTEN deficiency could underlie the neoplastic transformation of NSC. For this purpose, we first repressed CBP/p300 activity in *PTEN*^−/−^ NSCs by shRNA. Silencing CBP/p300 effectively decreased the migration of PTEN-deficient NSCs (Supplementary Fig. 14g,h). In addition, we compared the abilities of the WT, protein phosphatase-dead mutant (Y138L) and lipid phosphatase-dead PTEN mutant (G129E) of PTEN in rescuing the malignant phenotypes of *PTEN*^−/−^ NSCs. Although complementation with either the WT or the G129E mutant PTEN effectively rescued the neoplastic phenotypes in *PTEN*^−/−^ NSCs, mutation of PTEN at Y138L resulted in loss of rescue ability (Fig. 4l), which is consistent with its inability to dephosphorylate CREB (Supplementary Fig. 14i). These observations indicated that the neoplasm-suppressing activity of PTEN in NSCs is at least in part dependent on its ability of trans-repressing CREB/CBP.

Together, these data support a model that PTEN functions as a trans-repressor at the *PAX7* gene promoter in NSCs; PTEN deficiency relieves the repression and activates CREB/CBP-mediated transcription of *PAX7* during the neoplastic transformation of NSCs.

### The PTEN-PAX7 axis regulates malignant features of GBM

**Figure 5:**
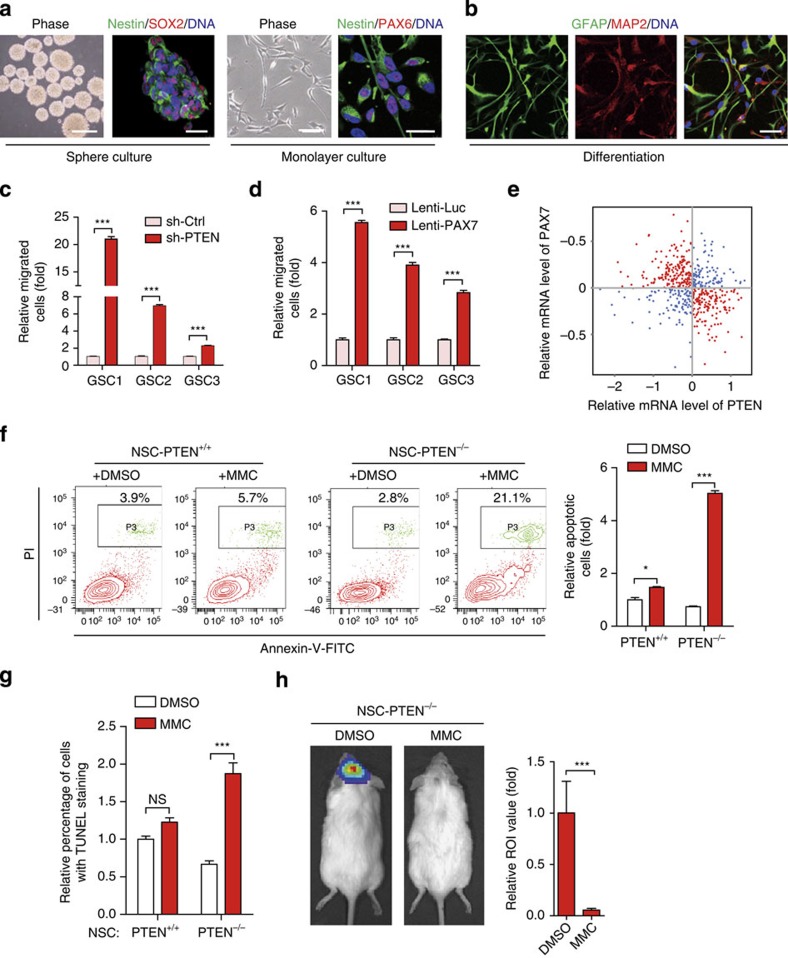
PTEN-PAX7 axis functions in GSCs and GBMs. (**a**,**b**) Immunofluorescence analysis of the indicated antigens in GSCs (**a**) and their spontaneously differentiated derivatives (**b**). Scale bars, 1 mm (phase) and 25 μm (IF). (**c**,**d**) Cell migration assay indicating increased cellular mobility in PTEN-knocked down (**c**) or PAX7-overexpressed (**d**) GSC lines. *n*=3. ***P<0.001 (*t*-test). (**e**) GBM microarray data were obtained from TCGA website and spearman correlation was calculated by all the samples which were plotted by red and blue dots. The gene expression level was log2 lowess normalized. PAX7 expression negatively correlated with PTEN (Spearman correlation coefficient=−0.28, *P* value=1.0 × 10^−11^). (**f**,**g**) FACS (**f**) and TdT-mediated dUTP nick end labelling (**g**) analyses of cell apoptosis in MMC (7.5 μM, 24 h) or vehicle-treated WT and *PTEN*^−/−^ NSCs. *n*=6. *P<0.05 (*t*-test); ***P<0.001 (*t*-test); NS, not significant. (**h**) Representative bioluminescent images of *PTEN*^−/−^ NSCs implanted animals in the presence or absence of MMC treatment are shown at day 70 after injection (left). Right panel indicates average bioluminescent signals. *n*=5 for dimethylsulphoxide (DMSO) treatment, and *n*=8 for MMC treatment. ***P<0.001 (*t*-test).

Our results support the view that human NSCs are the potential cell-of-origin for GBM, especially in the context of PTEN deficiency. It is perceivable that GSCs, the tumorigenic population within GBM, inherit genetic trait from mutated NSCs that potentially mediates the malignancy of GBM. We established three lines of GSCs from primary patient GBM specimens based on the established methods^30^,^47^. These GSCs exhibited features characteristic of NSCs and could be cultured under neurosphere or monolayer conditions (Fig. 5a,b, Supplementary Fig. 15a,b and Supplementary table 1)^30^. Western blotting and immunofluorescence showed that these GSCs expressed normal levels of PTEN protein (referred to as parental GSCs; Supplementary Fig. 15c). To create isogenic GSCs that are deficient for endogenous PTEN (referred to as GSCs-PTEN^low^), the parental GSCs were transduced with a lentiviral vector encoding PTEN shRNA. Silencing PTEN in the parental GSCs resulted in a marked increase of migration capability (Fig. 5c and Supplementary Fig. 15d). Correspondingly, GSCs-PTEN^low^ exhibited increased levels of phosphorylated AKT, phosphorylated CREB and PAX7 (Supplementary Fig. 15e,f). Knockdown of CBP/p300 in GSCs-PTEN^low^ not only impaired PAX7 expression but also compromised cellular migration potential (Supplementary Fig. 15g,h), whereas overexpression of *PAX7* in the parental GSCs resulted in a more aggressive migratory phenotype (the resulting cells referred to as GSCs-PAX7^high^), similar to that observed in GSCs-PTEN^low^ (Fig. 5d and Supplementary Fig. 15d,i).

To establish a connection between PTEN and PAX7 in human GBMs, we examined the Cancer Genome Atlas (TCGA) data set of 532 GBMs^48^, and found that GBMs with decreased PTEN expression demonstrated increased *PAX7* transcript (Spearman correlation coefficient=−0.28, *P* value=1.0 × 10^−11^; Fig. 5e), therefore providing a strong evidence that PTEN expression negatively correlates with *PAX7* transcripts in clinical GBM settings.

### Mitomycin C (MMC) triggers apoptosis in PTEN-deficient NSCs

Given that the PTEN deficiency is associated with increased metastasis, recurrence and drug resistance in GBM patients, discovery of compounds selectively killing PTEN-deficient GSCs may represent an important step forward towards precision therapy on GBM patients with known PTEN mutation(s). We therefore sought to utilize the isogenic NSC platform to screen compounds specifically kill PTEN-deficient NSCs while not the WT NSCs. By testing compounds with reported tumour-killing activity (Supplementary Table 2), we identified MMC, a clinically used anti-tumour agent with DNA-crosslinking activity^49^, as a potential candidate compound. MMC selectively killed *PTEN*^−/−^ NSCs while showed minimal effect on WT NSCs, as evidenced by propidium iodide staining and TdT-mediated dUTP nick end labelling assays (Fig. 4f,g and Supplementary Fig. 16a). Temozolomide, a clinically used anti-GBM drug, however, had a comparable effect on WT and *PTEN*^−/−^ NSCs (Supplementary Fig. 16b). MMC was also found cytotoxic to NSCs overexpressing *PAX7* (Supplementary Fig. 16c).

To evaluate the efficacy of MMC *in vivo*, we performed intracranial administration of MMC to mice harbouring neoplasm derived from *PTEN*^−/−^ NSC engrafts. MMC-treatment effectively repressed neoplastic growth from the *PTEN*^−/−^ NSC engrafts (Fig. 5h).

## Discussion

GBM is one of the most deadly forms of human cancers and has a median survival time of only ∼14.6 months. Up to date, most studies of GBM pathogenesis rely on GBM cell lines, primary GSCs or GBM cells from fresh specimens, or mouse GBM models. These conventional source materials have undoubtedly aided in our understanding of GBM pathogenesis and facilitated development of novel therapies, however, have their drawbacks. For instance, GBM or GSC lines bear significant variations from line to line, such as marker expressions, differences in cellular behaviours *in vitro* or *in vivo*, and sensitivities to drugs. These differences can be attributed to different culture conditions, pathological stages or, more likely, from their distinct genetic backgrounds. The complex genetic mutations, including the primary, secondary or evolved ones in tumour suppressors and oncogenes, as well as their interactions constitute a roadblock for studying the oncogenic identity and cell-of-origin for GSCs, as well as for aetiological understanding of GBM. Although it has been well established that GSCs are directly linked to NSCs, it has been challenging to study how genetic mutations may contribute to malignant transformation of NSCs as well as initiation and progression of GBM. The primary GBM subtype is acute and of high-grade and frequently harbours loss-of-function mutations in *PTEN* gene. Mouse models have been employed to study the role of PTEN deficiency in NSCs^13^,^50^,^51^. Given that species-difference exists between mouse and human, a human cellular platform to study GBM origin or pathogenesis associated with PTEN deficiency or other oncogenic events is thus urgently needed. It was not until very recently that two elegant human intestine tumour models generated using CRISPR/Cas9-based gene editing were reported^25^,^26^. Nevertheless, none of above-mentioned models investigated tumorigenesis in a genuine tumour-initiating niche. Our study for the first time uses genetically engineered human NSCs to study a specific genetic mutation on neoplastic transformation both *in vitro* and in intracranial tumour-initiating niches, and provided the proof-of-concept evidence that NSC is a most susceptible human adult stem cell type for PTEN-deficiency-induced oncogenic transformation. Nevertheless, it should be emphasized that GBM are frequently associated with more than one genetic mutations. Indeed, compared with the GSCs derived from clinically obtained GBM samples that potentially harbour multiple genetic mutations^52^, PTEN-deficient human NSCs show milder phenotypes in intracranial implantation assay, and do not show aggressive growth in non-neural microenvironment. We have shown that p53 insufficiency and PTEN deficiency resulted in a cooperative effect on NSC transformation, which is consistent with a recent mouse study indicating that simultaneous inactivation of Pten and p53 in mouse neural stem/progenitor cells led to malignant transformation and tumour formation^13^. From this point of view, the isogenic NSC platform also provides a useful model to study other individual neoplastic mutations, or their combinations in mediating malignant human NSC transformation.

Accumulating evidence indicated that PTEN plays a role in the nucleus through protein–protein interactions^27^,^44^. We showed here that PTEN is a nucleus-localized protein in human NSCs. It associates with and inactivates CREB/CBP at *PAX7* promoter, and mediates transcriptional repression of PAX7. To our knowledge, this is the first report to show that PTEN can trans-repress gene expression via the association with transcriptional activators on gene promoters. Notably, a recent report suggests that PTEN binds to Rad51 promoter and is required for its expression in MEFs^27^. In a separate study, PTEN is identified to be associated with histone H1 and accounts for maintenance of a condensed chromatin configuration^44^. Our findings, together with these observations, support the notion that nuclear PTEN may act as a key transcriptional regulator in a context-dependent manner. Given that *PAX7* is among the earliest genes involved in neural progenitor commitment in the embryo, the elevated expression of *PAX7* in PTEN-deficient GSCs may account for the low-differentiated histology of GBM, a common feature for more aggressive cancers. Together with the finding that PTEN is negatively correlated with PAX7 in clinical human GBM samples, it is likely that the overexpression of PAX7 in GSCs is one of the key factors mediating GBM tumorigenesis or malignancy.

Owing to its prevalence in GBM and other cancers, PTEN mutations have been regarded as a major target for drug discovery. Cell-based systems are advantageous for automate chemical screening or drug evaluation. Most of previous studies rely on cancer cells or primary cultures and use non-isogenic cells as control, which are limited by confounding effect of different genetic backgrounds. When it comes to screening for specific drug to kill GSCs, human fetal NSCs from a different genetic background are the common cells to be used as a control^53^. Our isogenic PTEN-knockout NSCs recapitulated certain features of GSCs, thus holding the potential to be used as a platform for screening ‘precision drugs' against GSCs based on ‘PTEN deficiency'. We found that MMC is a candidate compound, and preferentially targeting PTEN-deficient NSCs while sparing their PTEN-proficient counterparts. We further showed that transcranial injection of MMC could diminish neoplasia from PTEN-deficient NSCs. Nevertheless, although MMC is used in the clinic for other cancers, it does not cross the blood–brain barrier and has not been used for GBM. Thus, developing advanced MMC derivatives being capable of passing through blood–brain barrier and with reduced toxicity may represent a potential therapeutic strategy for GBM harbouring *PTEN* mutations, despite that the underlying pharmacological mechanism warrants further investigations. To our best knowledge, this is the first study using isogenic NSCs bearing a genetically engineered carcinogenic mutation as a potential drug discovery strategy. We propose here a simplified approach to examine ‘one mutation' at a time, using human ESC (or iPSC)-derived NSCs with a well-known genetic background to screen or evaluate drugs being capable of targeting certain genetic mutations.

## Methods

### Antibodies and reagents

The following commercial primary antibodies were used: anti-PTEN (9559; Western blotting (WB),1:1,000; immunofluorescence (IF), 1:200), anti-Phospho-PTEN (pSer380, 9551, 1:1,000), anti-Phospho-AKT (pSer473, 4060, WB,1:1,000;IF, 1:200), anti-Phospho-AKT (pThr308, 2965, 1:1,000), anti-Phospho-GSK-3β (pSer9, 5558, 1:1,000), anti-Phospho-mTOR (pSer2448, 5536, 1:1,000), anti-phospho-CREB (pSer133, 9198, 1:1,000), anti-CREB (9197, 1:1,000) antibodies were from Cell Signaling Technology; anti-NANOG (ab21624, 1:250), anti-Ki67 (ab16667, 1:1,000), anti-Nucleolin (ab22758, 1:200), anti-CBP (ab2832, 1:1,000) antibodies were from Abcam; anti-OCT4 (sc-5279, 1:100), anti-SOX2 (sc-17320, 1:100), anti-Actin (sc-130301, 1:2,000), anti-GFAP (sc-9065, 1:1,000), anti-TRA-1-81 (sc-21706, 1:20) antibodies were from Santa Cruz Biotechnology; anti-Nestin (MAB5326,1:500), anti-Human nuclei (MAB1281, 1:1,000) antibodies was from Millipore; anti-MAP2 (m4403, 1:500), anti-Tuj1 (T2200, 1:500), anti-Flag (M2, 1:2,000), anti-SMA (A5228, 1:100), anti-AFP (A8452, 1:200), anti-tubulin (T5168, 1:2,000) antibodies were from Sigma; anti-CD34 (555822, 1:50), anti-CD43 (560198, 1:50), anti-CD45 (555482, 1:50), anti-CD73 (550741, 1:50), anti-CD90 (555595, 1:100) antibodies were from BD Biosciences; anti-CD105 (17–1057, 1:100) antibody was from eBioscience; anti-PAX6 (PRB-278P, 1:500) antibody was from Covance; anti-PAX7 antibody (WB, 1:500; IF, 1:50) was from Atsushi Kawakami; anti-CD133 (130-080-801, 1:10) was from Miltenyi Biotec; MMC was purchased from Biomol International. Temozolomide was purchased from Sigma.

### Cell culture

H9 ESCs were purchased from WiCell Research Institute and cultured as previously described^54^. Briefly, human fibroblast-derived iPSCs, H9 ESCs and their *PTEN* knockout derivatives were maintained on MMC inactivated MEFs in human ESC culture medium; whereas the feeder-free cultures were maintained on Matrigel- (BD Biosciences) coated plates in mTeSR medium (STEMCELL Technologies). The human ESC culture medium contains: 80% DMEM/F-12 (DMEM; Invitrogen), 20% knockout serum replacement (Invitrogen), 1% GlutaMAX (Invitrogen), 1% non-essential amino acids (Invitrogen), 1% Penicillin–Streptomycin (PS, Invitrogen), 55 μM β-mercaptoethanol and 10 ng ml^−1^ of human basic fibroblast growth factor (bFGF; Joint Protein Central). MSCs were grown on Gelatin-coated plates in culture medium containing 90% α-MEM (Invitrogen), 10% fetal bovine serum (AusGeneX), 1% non-essential amino acid, 1% PS and 1 ng ml^−1^ of bFGF. NSCs were cultured in Neural Stem cell Maintenance Medium containing 50% Advanced DMEM/F12, 50% Neurobasal, 1 × N2, 1 × B27, 2 mM GlutaMAX, 10 ng ml^−1^ of human leukemia inhibitory factor (hLIF), 3 μM CHIR99021 and 2 μM SB431542. GSCs were grown in medium containing 90% Neurocult NSC Basal Medium (STEMCELL Technologies), 10% NeuroClut NSC Proliferation Supplements (STEMCELL Technologies), 20 ng ml^−1^ of EGF and 20 ng ml^−1^ of bFGF and 2 μg ml^−1^ of heparin^55^. HEK293T cells were maintained in high-glucose DMEM containing 10% fetal bovine serum (FBS, Gemini). No mycoplasma contamination was observed during cell culture.

### Generation of PTEN knockout (*PTEN* ^−/−^) human ESCs

TALENs against the human *PTEN* gene (*PTEN*-TALEN_Left and *PTEN*-TALEN_Right) were purchased from Addgene (Plasmid #36764 Plasmid #36765). The donor plasmid for HR was described previously^22^. The following PCR primers were used for cloning the homology arms into the donor vector (*PTEN*-HR-Neo-pCR2.1):

Left-F: 5′- CGGGGTACCGACCGTCCCTGCATTTCCCTCTACACT -3′;

Left-R: 5′- CGCGGATCCCGGAATGGGGAGAAGACGAATAATCCTCCGAAC -3′;

Right-F: 5′- ACGCGTCGACCCTGGTTGCAAGTGTCAA GCCACCGAT -3′;

Right-R: 5′- ATAAGAATGCGGCCGCCCCCATCCCTAATCAAAACCAATGTGTTGTACCTG -3′;

H9 ESCs were pre-treated with ROCK inhibitor Y-27632 (Sigma) 24 h before plasmid electroporation. On the day of electroporation, H9 ESCs were individualized with TrypLE (Invitrogen) and filtered through a 40-μm cell strainer to remove cell clumps. For TALEN-mediated engineering of *PTEN*^−/−^ ESC lines, 10^6^ cells were counted and resuspended in 1 ml of MEF-conditioned medium containing 10 μM ROCK inhibitor Y-27632, then mixed with plasmids (8 μg *PTEN*-TALEN_Left, 8 μg *PTEN*-TALEN_Right, 2 μg Neo-*PTEN*-HR-pCR2.1 donor vector) and electroporated. After electroporation, cells were plated on MMC inactivated G418-resistent MEF cells. Two days later, cells were treated with 50 μg ml^−1^ of G418 (Gibco) for selection. After 14–21 days of selection, G418-resistant clones were manually picked and transferred to 96-well plates and expanded for genotyping. Gene-targeting was determined by PCR-screening of genomic DNA from the drug-resistant clones with the following primers:

P1: 5′- AGGCTGTTACAGTCAAATCTCTGCGAACGAT -3′;

P2: 5′- CCCCAAAGGCCTACCCGCTTCCATTGCTCA -3′;

P3: 5′- CTACCTGCCCATTCGACCACCAAGCGAAACATC -3′;

P4: 5′- TGTCCACTGCCACAATTCGCATTACCAAACTCA -3′.

Homozygous deletion of exon 1 of the *PTEN* gene was verified by a two-step PCR with the following primers:

P5: 5′- CCCAGACATGACAGCCATCATCAAAGAGATCG -3′;

P6: 5′- AAAACGGTGAGCAGCAATTCTGGCATCACA -3′;

P7: 5′- TTTGGGCCCCACACAGTGCAAGCTAAAGCAACCCCGTCT -3′;

P8: 5′- CCGCTCGAGAACATCACTGGTCCTGGGCAAAATCGAGA -3′.

The *PTEN* locus was further examined by Southern blotting after extracting genomic DNA from PCR-positive clones. The neomycin-resistance cassette was removed according to the previously described method^22^,^23^. Briefly, in order to remove the neomycin-resistance cassette, *PTEN* knock-out hESCs (5 × 10^6^ cells) were electroporated with pCAG-Flpo-2A-puro vector (10 μg), and then plated on MMC-inactivated DR4-MEF cells. Two days later, puromycin (1 μg ml^−1^; Invitrogen) was added to the medium to enrich Flpo recombinase-expressed cells. The emerging colonies were expanded, and the removal of the neomycin-resistance cassette was verified by PCR using the following primers: P1: 5′- AGGCTGTTACAGTCAAATCTCTGCGAACGAT -3′ and P4: 5′- TGTCCACTGCCACAATTCGCATTACCAAACTCA -3′.

### Flow cytometry analysis

For TRA-1-81 analysis, each 100 μl of cell suspension (10^6^ cells) was used. Cells were incubated with the primary antibody mouse anti-TRA-1-81 (Santa Cruz) and then the secondary Alexa Fluor 568-labelled Goat anti-Mouse IgM (Invitrogen). For CD133 analysis, each 100 μl of cell suspension (10^6^ cells) was used. Cells were incubated with the primary antibody CD133/1(AC133)-PE (Miltenyi Biotec). For cell cycle analysis, cells were processed with the Click-iT EdU Flow Cytometry Assay Kits (Invitrogen) according to the manufacturer's instructions. Briefly, the cells were harvested after incubation with EdU (10 μM) in medium. 100 μl of cell suspension (10^6^ cells) for each sample were used. For the detection of cell apoptosis, each sample (10^6^ cells) was subjected to Annexin V-PE staining^20^. Cells were examined by fluorescence-activated cell sorting (FACS) using a flow cytometer (FACSort; Becton, Dickinson and Company), and the cell cycle populations were determined using ModFit software (Verity Software House, Inc.).

### Generation and characterization of NSCs

Neural induction was based on previous reports^20^,^22^. In brief, ESCs cultured on MEF feeder cells were challenged with NIM-1 medium (50% Advanced DMEM/F12 (Invitrogen), 50% Neurobasal (Invitrogen), 1 × N2 (Invitrogen), 1 × B27 (Invitrogen), 2 mM GlutaMAX (Invitrogen) and 10 ng ml^−1^ of hLIF (Millipore), 4 μM CHIR99021 (Cellagentech), 3 μM SB431542 (Cellagentech), 2 μM Dorsomorphin (Sigma) and 0.1 μM Compound E (EMD Chemicals Inc.). Two days later, medium was switched to NIM-2 (50% Advanced DMEM/F12, 50% Neurobasal, 1 × N2, 1 × B27, 2 mM GlutaMAX, 10 ng ml^−1^ of hLIF, 4 μM CHIR99021, 3 μM SB431542 and 0.1 μM Compound E) for additional 5 days. The cultures were then split onto Matrigel-coated plates and maintained in neural stem cell maintenance medium (NSMM) containing 50% Advanced DMEM/F12, 50% Neurobasal, 10 ng ml^−1^ of hLIF, 1 × N2, 1 × B27, 2 mM GlutaMAX, 3 μM CHIR99021 and 2 μM SB431542.

### Neuronal differentiation assay

1 × 10^5^ NSCs cultured on Matrigel-coated one well of six-well plate were switched to neuronal differentiation medium containing DMEM/F12, 1 × N2, 1 × B27, 200 μM Ascorbic acid (Sigma), 400 μM dbcAMP (Sigma), 10 ng ml^−1^ of GDNF (Peprotech) and 10 ng ml^−1^ of BDNF (Peprotech). Laminin (Sigma) was used to facilitate differentiation. Cells were cultured for 21 days, and then characterized by MAP2 and Tuj1 immunostaining^20^,^22^.

### Bisulfite sequencing of the *OCT4* promoter

Bisulfite conversion of DNA was carried out using CpGenome Fast DNA Modification Kit (Millipore) following the manufacturer's recommendation. About 1 μg of DNA was used as starting material. A genomic fragment of the *OCT4* promoter was amplified using LA Taq Hot Start Version (TAKARA) as previously described^56^. In brief, PCR products were purified by gel extraction using QIAquick columns (Qiagen), and subsequently cloned into the pEasy-T1 vector (Transgen). Thirteen clones from each sample were sequenced with the universal primer M13.

### Derivation of MSCs from ESCs

MSCs were differentiated from ESC lines as previously described^22^,^23^. In brief, groups of 10–14 EBs were plated on Matrigel-coated six-well plates in α-MEM (Invitrogen) medium with 10% FBS (Hyclone), 1% PS, 10 ng ml^−1^ of bFGF and 5 ng ml^−1^ of TGFβ (HumanZyme). Cells were left for about 10 days until confluent MSC-like populations occurred. The resulting cells were passaged once and then analysed and sorted by FACS using various antibodies related to the MSC signatures. The cells after sorting were set to passage 0 (P0). Expressions of MSC-specific markers were verified by immunostaining with antibodies against CD73 (BD), CD90 (BD) and CD105 (eBioscience).

### Isolation and culture of human GSCs

Three glioblastoma samples were obtained in the Beijing Tiantan Hospital, following approval from the institutional review board, and written informed consent was obtained from all patients. GSCs were isolated according to previously described methods with minor modifications^30^,^55^. Briefly, tumour tissues were dissociated into single cells with Accumax Cell Dissociation Solution (Millipore) for 30 min at 37 °C. 2 × 10^6^ single cells were initially seeded into one well of a six-well plate to allow formation of spheres/aggregates. In addition to sphere culture, GSCs were also cultured as monolayer in Matrigel-coated culture dishes. Cells were stably maintained either as spheres or as monolayer culture.

### Neurosphere assay

2 × 10^4^ single NSCs were seeded into one-well of an ultra-low attachment 96-well plate (Corning) to allow neurosphere formation. Medium was changed every two days. The diameters of neurospheres were measured using Image J.

### Luciferase reporter assays

A 981-bp genomic DNA fragment containing the PTEN docking site upstream of the TSS of *PAX7* was cloned into the pGL3-Promoter vector (Promega). The DNA fragment was amplified by PCR using the following primers: 5′- CGGGGTACCCAAGTCTGGCCCAGCCCACACACTCAG -3′ (forward primer); 5′- CCGCTCGAGGCCCTCCCCCGGGAGAGATTGGGAGATC -3′ (reverse primer). Transient transfection with the luciferase reporter plasmids was performed in 12-well cell culture plates according to the manufacturer's protocol. Cells were lysed 48 h post transfection, and firefly and Renilla luciferase activity were measured with a Synergy H1 Hybrid Reader(BioTek), and Renilla was used as an internal control.

### Immunofluorescence microscopy

Cells were fixed with 4% formaldehyde in PBS at room temperature (RT) for 20–30 min. After fixation, cells were permeabilized with 0.4% Triton X-100 in PBS for 5 min at RT. After incubation with 10% FBS or BSA in PBS for 30 min, cells were incubated at RT for 1 h or at 4 °C overnight with the primary antibody. Subsequently, incubation at RT for 20 min with the corresponding secondary antibody was carried out. Nuclei were stained with Hoechst 33342 (Invitrogen). A Leica SP5 confocal was used for Immunofluorescence microscopy.

### Teratoma analysis

Teratoma assay was performed as described^57^. In brief, 3 × 10^6^ ESCs were administrated subcutaneously into NOD/SCID mice (male, 6–8 weeks). Mice were killed 6–12 weeks after injection, and teratomas were analysed by immunofluorescence staining. All animal experiments were conducted with approval of the Institute of Biophysics, Chinese Academy of Science.

### SA-β-Gal staining

Staining was conducted as described previously^23^. In brief, cells were fixed at RT in 2% formaldehyde and 0.2% glutaraldehyde for 3 min. Fixed cells were then stained with fresh staining solution at 37 °C overnight. The percentage of cells positive for SA-β-Gal signal were quantified and statistically analysed.

### Lentivirus production

Expression and purification of lentiviruses were performed as previously described with minor modifications^23^. For generation of lentiviral vectors encoding shRNA targeting PTEN, PAX7 or CBP/p300, corresponding shRNA oligos (Supplementary Table 3) were cloned into the pLVTHM vector pre-cleaved by ClaI and MluI. For lentiviral vectors expressing PTEN, PAX7 or luciferase (control), corresponding cDNAs were cloned into the pLE4 vector (a kind gift from Dr Tomoaki Hishida). For lentiviral packaging, HEK293T cells were co-transfected with lentiviral expression or shRNA vectors, as well as psPAX2 (Addgene, 12260) and pMD2.G (Addgene, 12259). Lentiviral particles were collected after 48 h and concentrated by ultracentrifugation at 19,400 g for 2.5 h. Concentrated viruses were used for titre determination and cell transduction.

### Western blotting, IP and RT-qPCR

For Western blotting, cells were lysed in RIPA buffer (25 mM Tris-HCl pH 7.6, 150 mM NaCl, 1% NP-40, 1% sodium deoxycholate, 0.1% SDS) with protease inhibitor cocktail (Roche). A BCA kit (Thermo Fisher Scientific) was used for protein quantification. About 20 μg of protein lysate was subjected to SDS–PAGE electrophoresis and electrotransferred to a PVDF membrane (Millipore). Then primary and HRP-conjugated secondary antibodies were used to visualize the target proteins. The quantification of western blot was performed with Image Lab software for ChemiDoc XRS system (Bio-Rad). For IP, ∼1 × 10^7^  cells was lysed on ice for 1 h in NP40 lysis buffer (50 mM Tris-HCl, pH 8.0, 1% NP-40, 150 mM NaCl, 2 mM EDTA, 1 mM PMSF, Complete EDTA-free Protease Inhibitor Cocktail). After adjusting protein concentrations to about 4 mg ml^−1^, inputs were aliquoted, and lysates were incubated with anti-Flag agarose (Sigma) overnight at 4 °C. After three washes in NP-40 lysis buffer, bound proteins were eluted with 1 × SDS loading buffer, and samples were analysed by western blotting with the indicated antibodies. For RT–qPCR, cellular total RNA was extracted by TRIzol (Invitrogen), and genomic DNA was removed by DNA-free Kit (Ambion), followed by cDNA synthesis with GoScript Reverse Transcription System (Promega). RT–qPCR was performed using iTaq Universal SYBR Green Supermix (Bio-Rad). Primer sequences are given in Supplementary Table 3. The uncropped images of the western blots and gels are shown in Supplementary Fig. 17.

### Cell viability assay

Cell viability was determined with CellTiter 96 AQueous One Solution Cell Proliferation Assay MTS (3-(4,5-dimethylthiazol-2-yl)-2,5-diphenyltetrazolium).

### *In vitro* cell migration assay

The cell migration of NSCs, MSCs and GSCs through the Matrigel (BD Bioscience) *in vitro* was performed as previously described^58^. First, the top chamber of the Transwells (24-well insert; pore size, 8 mm; Corning Costar) pre-coated with Matrigel was seeded with 2.5 × 10^4^ cells in 100 μl basal medium (50% Advanced DMEM/F12, 50% Neurobasal for hNSCs, αMEM for hMSCs and GSC culture medium without EGF, FGF2 and Heparin for GSCs). The corresponding complete culture medium for each cell type was put into the bottom chambers. Cells that migrated through the Transwells were stained and counted.

### Single-cell clonal expansion assay

To determine the clonal expansion efficiency of NSCs and MSCs, 2,000 cells were seeded in a Matrigel-coated 12-well plate, and cultured until clear cell colonies formed. The relative colony number was then determined by crystal violet staining.

### Metabolomics evaluation

Metabolite profiling was carried out at Metabolon with LC-MS and GC–MS methods as described previously^59^. Briefly, 10^6^ ESCs (*PTEN*^*+/+*^ versus *PTEN*^−/−^) and NSCs (*PTEN*^*+/+*^ versus *PTEN*^−/−^) were pelleted, and five replicates were stored at −80 °C. Samples were prepared and analysed by using Metabolon's standard solvent extraction methods. 100 μl of each sample was extracted by methanol. After centrifugation, the supernatant consisting of metabolites was used for MS and LC-MS analyses. The extracts were equally split into two parts for analysis on LC/MS/MS (Thermo Fisher linear Ion Trap mass spectrometer with a Fourier Transform) and GC/MS (Mat-95 XP mass spectrometer) platforms. Data were analysed with Welch's two-sample *t*-test.

### Lactate measurements

Lactate secreted in the culture medium was collected and measured by a colorometric L-Lactate assay kit (Abcam) following the manufacturer's instructions.

### Mouse brain *in vivo* imaging

Before intracranial implanting, NSCs were transduced with lentivirus expressing luciferase. 2 μl of a 1 × 10^7^ cells per ml cell suspension in high-glucose PBS were injected to the hemi-striatum using stereotactic injection. The following coordinate parameters were used: antero-posterior=0; medio-lateral=+2.5 mm; dorso-ventral=−3.5 mm. About 10 weeks post injection, mice were injected into peritoneal cavity with luciferin to trace tumour cells *in vivo*. The animals were next anaesthetized, and bioluminescence was imaged using the IVIS Lumina system (Perkin-Elmer). Images were taken every 2 min for a course of 30 min (10 s exposure per image). The mice brains were also imaged 1 and 2 h post injection (10 s exposure per image). Images were processed using Living Image software. Regions of interest were drawn based on each cell mass, and the total number of photons was recorded for each region of interest. Immunofluorescence staining and H&E staining were carried out on 5 μm cryostat sections.

### Magnetic resonance imaging

MRI for mouse brain was performed on a Bruker's PharmaScan 7 T MRI imaging system. The system is equipped with standard actively shielded gradient system. A 72-mm birdcage resonator was used for excitation, and detection was done with a 30-mm surface coil. T2-weighted images were consecutively acquired by using a rapid-acquisition relaxation enhanced sequence. Animals were anaesthetized before MRI.

### RNA isolation and sequencing library construction

A total of 5–10 × 10^5^ cells were used to isolate RNA with RNeasy Mini Kit (Qiagen). 1.5–2 μg of RNA was qualified by Fragment Analyzer (Advanced Analytical) and was used to construct sequencing libraries using the TruSeq RNA Sample Preparation Kit (Illumina) following the manufacture's protocol.

### ChIP and sequencing

ChIP-seq was done following a previous protocol with minor modifications^60^. Briefly, cells were cross-linked by 1% (vol/vol) formaldehyde for 8 min at RT and then quenched by 125 mM glycine for 5 min at RT. Then, the cells were lysed in lysis buffer (50 mM Tris-HCl, 10 mM EDTA, 1% SDS, pH8.0), which was followed by chromatin shearing using a Covaris S2 instrument. The samples were then incubated overnight with Protein A-bead bound with the following antibodies separately: anti-PTEN (CST, 9559), anti-CBP (Abcam, ab2832), anti-phospho-CREB (CST, 9198) anti-H3K4me3 (CST, 9751, s) and anti-H3K27me3 (CST, 9733). Normal rabbit IgG (Santa Cruz, SC-2027) or input was used as a negative control. To obtain the DNA of interest, chromatin with the target modification was eluted in elution buffer (20 mM Tris-HCl, 5 mM EDTA, 50 mM NaCl, pH7.5), and then incubated with Proteinase K on a thermomixer at 1,300 r.p.m. for 2 h at 68 °C. Next, the DNA was extracted and purified via phenol–chloroform–isoamyl alcohol extraction and ethanol precipitation. Samples for sequencing including H3K4me3, H3K27me3 and IgG were constructed into libraries with NEBNext DNA Library Prep Reagent Set for Illumina (NEB) as described by the manufacturer.

### ChIP-PCR mapping of PTEN-associated genomic region

We mapped the PTEN-binding site at PAX7 promoter spanning from −5,000 to +3,251 with ChIP-PCR using different primer sets. In brief, to obtain the DNA region of interest, lysed samples were incubated overnight with Protein A-bead bound to anti-PTEN antibody. Then, extracted DNAs were subjected to PCR amplification of the different genomic regions by using different primer sets (Supplementary Table 3).

### Whole genome sequencing

Genomic DNA was extracted from 1 × 10^6^ cells by DNeasy Blood & Tissue Kit (Qiagen) and sheared into 150–200 bp fragments by Covaris S2 and the quality was analysed via Fragment Analyzer (Advanced Analytical). The sequencing libraries were constructed using NEBNext DNA Library Prep Reagent Set for Illumina (NEB).

### RRBS and TA-RRBS

RRBS and TA-RRBS were performed as we previously reported^61^. For RRBS, after digested with *Msp*I, genomic DNA was size-selected and spiked in with 0.5% fully methylated lambda DNA. DNA was then treated with bisulfite after ligation with pre-methylated adapters and amplified with PfuTurbo Cx Hotstart DNA polymerase (Agilent Technologies). For TA-RRBS, after *Msp*I digestion, 50–600 bp DNA fragments were first gel-selected, and then glucosylated with β-glucosyltransferase and oxidized by recombinant mouse Tet1 (mTet1) subsequently. The final TA-RRBS libraries were generated after bisulfite treatment and amplification with PfuTurbo Cx Hotstart DNA polymerase as stated above.

### RNA-Seq data analysis

We aligned reads from RNA-Seq using TopHat^62^ and Cufflinks^63^ and differentially expressed genes were analysed as previously described^64^. A total of 14,797, 14,782, 14,030 and 14,322 genes with FPKM>0.1 were detected in *PTEN*^*+/+*^ NSC, *PTEN*^−/−^ NSC, *PTEN*^*+/+*^ MSC and *PTEN*^−/−^ MSC, respectively. 281, 705 genes (*q*-value<0.05, *P*-value<0.05, FC>2) were significantly downregulated and 326, 1,139 genes (*q*-value<0.05, *P*-value<0.05, FC>2) were significantly upregulated in *PTEN*^−/−^ NSCs and MSCs, respectively. GO for enrichment of genes was assessed by DAVID^65^.

### ChIP-seq data analysis

Sequencing reads from the ChIP-Seq experiment were mapped to the human reference genome (hg19) by Burrows-Wheeler Aligner (BWA)^66^, retaining only unique non-duplicate reads without mismatches in the first 15 bp. Peaks were then called by MACS by default parameters, using IgG as control^67^. Histone modification level was defined as non-duplicate reads in peaks (PR) normalized by total non-duplicate reads (NR), *D*=PR/NR. Modification level variation (*PTEN*^−/−^ versus *PTEN*^*+/+*^>2) was assessed by the following command: macs2 bdgdiff –t1 k4wt.bdg –t2 k4ko.bdg –c1 k4wt_control_lambda.bdg –c2 k4ko_control_lambda.bdg –d1 6191314 –d2 6191314 –outdir./ −C 0.3 –g 200 –l 50.

### CREB-binding sites analysis

Promoter for CREB-binding sites analysis was defined as 3 kb upstream of TSS and 300 bp downstream of TSS. CREB-binding sites with *P*-value less than 1E-4 among the promoter regions were found by FIMO^68^ using CREB motif downloaded from MEME database (MEME version 4.4).

### Genome-wide CNV analysis

Reads from whole-genome DNA sequencing were mapped to the human reference genome (hg19 from UCSC) by BWA. Signal of 500 kb window CNV analysis was calculated by the following formula, *C*=5*d*10^9^/((500,000-*t*)*b), in which *d* is the base number in 500 kb that mapped to the reference, *t* is the number of N in the 500 kb window and *b* is the total bases that mapped to the reference.

### RRBS and TA-RRBS data analysis

First, sequencing reads were trimmed using trim_galore_v0.3.3 by the following command, trim_galore --quality 20 --phred33 --stringency 3 --gzip --length 50 --rrbs --paired --trim1 --output_dir $out $raw1 $raw2. Then trimmed reads were mapped to the hg19 reference genome by Bismark^69^ and Bowtie^70^ using default parameters. Levels of 5hmC were determined by C/C+T of TA-RRBS and 5mC were determined by (C/(C+T)−5hmC%). In our study, only genome regions with coverage >=5 were taken into account.

### Statistical analysis

Data were analysed with Student's *t*-tests and were presented as mean±standard deviation (s.d. or s.e.m.). *P*<0.05 was considered as significant.

## Additional information

**Accession codes**: The RNA-seq, ChIP-seq, RRBS, TA-RRBS sequencing data sets have been deposited in NCBI Gene Expression Omnibus (GEO) under accession code GSE61794. And the whole-genome DNA sequencing data for CNV analysis are deposited to the Sequence Read Archive (SRA) under accession number of SRP048488.

**How to cite this article**: Duan, S. *et al*. PTEN deficiency reprogrammes human neural stem cells towards a glioblastoma stem cell-like phenotype. *Nat. Commun.* 6:10068 doi: 10.1038/ncomms10068 (2015).

## Supplementary Material

Supplementary Information Supplementary Figures 1-17, Supplementary Tables 1-3 and Supplementary References

Supplementary Data 1 Metabolomics evaluation in ESCs (PTEN+/+ vs PTEN-/-) and NSCs (PTEN+/+ vs PTEN-/-)

Supplementary Data 2 RNA-seq and epigenome analyses NSCs (PTEN+/+ vs PTEN-/-) and MSCs (PTEN+/+ vs PTEN-/-)

Supplementary Data 3 RNA-seq analysis in WT and PAX7-overexpressed NSCs

Supplementary Data 4 Potential CREB-targets and genes with increase of H3K4me3 among the 326 upregulated genes in PTEN-/- NSCs
