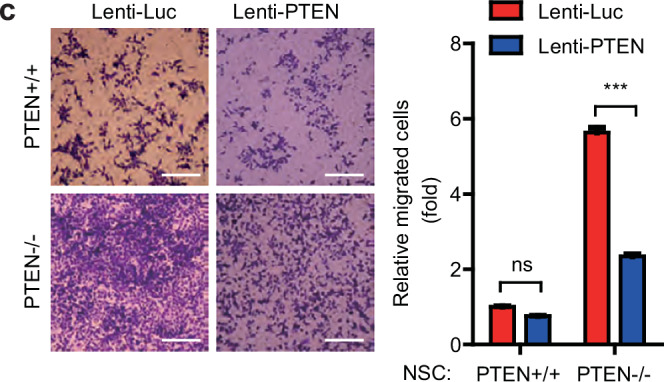# Author Correction: PTEN deficiency reprogrammes human neural stem cells towards a glioblastoma stem cell-like phenotype

**DOI:** 10.1038/s41467-025-60996-8

**Published:** 2025-06-20

**Authors:** Shunlei Duan, Guohong Yuan, Xiaomeng Liu, Ruotong Ren, Jingyi Li, Weizhou Zhang, Jun Wu, Xiuling Xu, Lina Fu, Ying Li, Jiping Yang, Weiqi Zhang, Ruijun Bai, Fei Yi, Keiichiro Suzuki, Hua Gao, Concepcion Rodriguez Esteban, Chuanbao Zhang, Juan Carlos Izpisua Belmonte, Zhiguo Chen, Xiaomin Wang, Tao Jiang, Jing Qu, Fuchou Tang, Guang-Hui Liu

**Affiliations:** 1 National Laboratory of Biomacromolecules, Institute of Biophysics, Chinese Academy of Sciences, Beijing, 100101 China; 2 Biodynamic Optical Imaging Center, College of Life Sciences, Peking University, Beijing, 100871 China; 3 FSU-CAS Innovation Institute, Foshan, 528000 China; 4 State Key Laboratory of Stem Cell and Reproductive Biology, Institute of Zoology, Chinese Academy of Sciences, Beijing, 100101 China; 5 Department of Pathology, Carver College of Medicine, University of Iowa, Iowa City, Iowa 52242 USA; 6 Gene Expression Laboratory, Salk Institute for Biological Studies, 10010 North Torrey Pines Road, La Jolla, California 92037 USA; 7 Department of Molecular and Cellular Physiology, Stanford University School of Medicine, Stanford, California 94305 USA; 8 Universidad Cato´lica San Antonio de Murcia (UCAM) Campus de los Jero´nimos, No 135 Guadalupe, 30107 Murcia, Spain; 9 Research Center for Translational Medicine, Shanghai East Hospital, School of Life Sciences and Technology, Tongji University, Shanghai, 200092 China; 10 Beijing Institute for Brain Disorders, Beijing, 100069 China; 11 Cell Therapy Center, Xuanwu Hospital Capital Medical University, Beijing, 100053 China; 12 Ministry of Education Key Laboratory of Cell Proliferation and Differentiation, Beijing, 100871 China; 13 Center for Molecular and Translational Medicine, CMTM, Beijing, 100101 China; 14 Peking-Tsinghua Center for Life Sciences, Peking University, Beijing, 100871 China

Correction to: *Nature Communications* 10.1038/ncomms10068, published online 03 December 2015

In the version of the article initially published, in Fig. 2c, the representative image for NSC-PTEN^+/+^ Lenti-Luc was inadvertently sourced from another experimental condition during figure preparation. The error was in presentation only and does not affect the conclusions or results of the study. The corrected image appears as Fig. 1 below. This amendment serves to correct the article.

Fig. 1 Corrected Fig. 2c