# Supplementary material for: PTEN deficiency reprogrammes human neural stem cells towards a glioblastoma stem cell-like phenotype
Source: Nat Commun. 2015 Dec 3;6:10068. doi: 10.1038/ncomms10068 (PMC4686761; doi:10.1038/ncomms10068)
Supplement: Supplementary Information — Supplementary Figures 1-17, Supplementary Tables 1-3 and Supplementary References [file ncomms10068-s1.pdf]

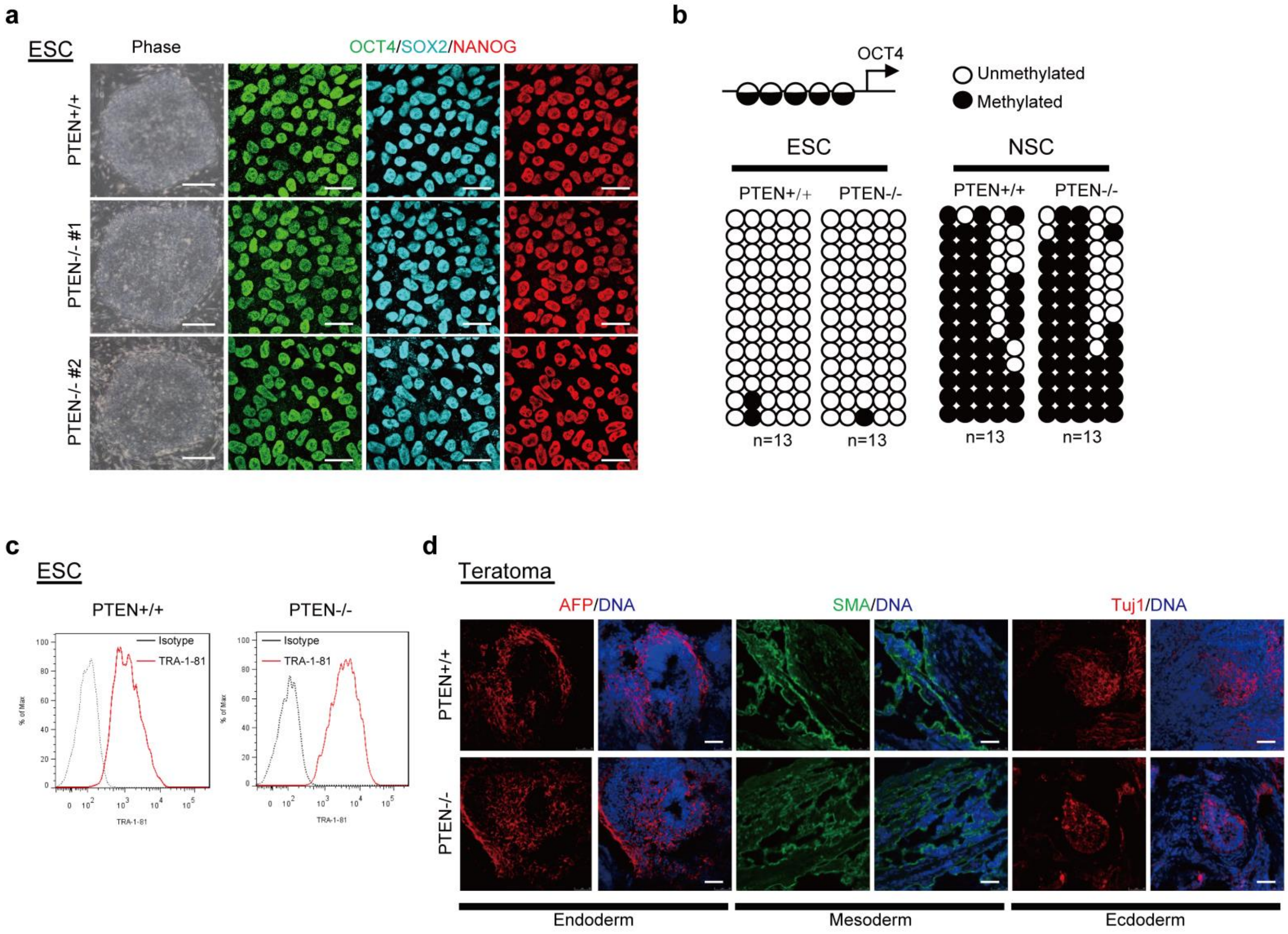


**Supplementary Figure 1. Characterization of PTEN knockout ESCs.** (a) Morphology and immunofluorescence analysis of the pluripotency markers in WT and *PTEN*$^{-/-}$ ESCs (line #1 and line #2). Nuclei were stained with Hoechst 33342. Scale bars, 1mm (phase) and 25 μm (IF). (b) DNA methylation status of the *OCT4* promoter in WT and *PTEN*$^{-/-}$ ESCs and NSCs. (c) FACS analysis of TRA-1-81 in WT and *PTEN*$^{-/-}$ ESCs. (d) Immunostaining of representative markers of three germ layers in teratomas developed from WT and *PTEN*$^{-/-}$ ESCs. Scale bars, 50 μm.

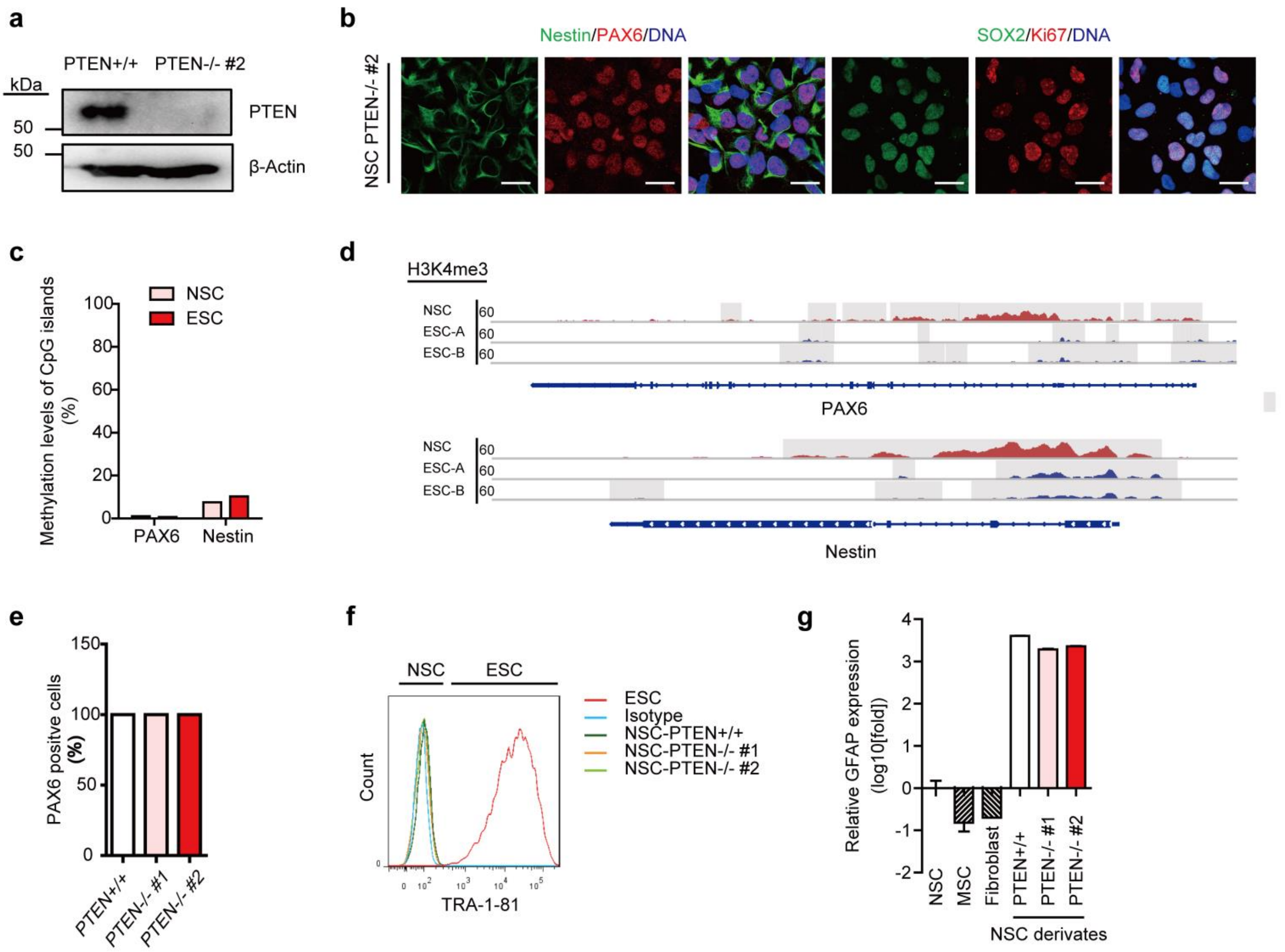


**Supplementary Figure 2. Characterization of PTEN knockout NSCs.** (a) Immunoblotting verified the absence of PTEN protein in *PTEN*$^{-/-}$ NSC (line #2) with an anti-PTEN antibody. β-Actin was used as loading control. (b) Immunostaining of neural progenitor specific markers in *PTEN*$^{-/-}$ NSC (line #2). Scale bars, 25 μm. (c) Methylation analysis shows comparable methylation level at CpG islands within PAX6 and Nestin Promoter in NSCs and ESCs. Methylation levels of CpG islands (CGIs) of Nestin and PAX6 in both NSCs and ESCs (The human ESCs data were downloaded from GEO database (GSM1493983, GSM1493984, GSM1493985)). The average methylation levels for Nestin and PAX6 in NSC are 1.0% and 7.5% covering 83 CpG sites and 354 CpG sites, respectively. For ESC, the average methylation level for CGIs of Nestin and PAX6 is 0.6% and 10.3% of these 83 CpG sites and 354 CpG sites, respectively. The result showed that the CGIs of Nestin and PAX6 are hypomethylated in both NSCs and ESCs. (d) H3K4me3 signal tracks in NSCs and ESCs at the loci of PAX6 and Nestin, two NSC specific markers, respectively. (The human ESCs data were downloaded from GEO database (SRR067951 for ESC-A; SRR179703 for ESC-B). (e) PAX6 positive cells were counted by analyzing acquired digital IF images. 100% of WT and *PTEN*$^{-/-}$ NSCs are positive for PAX6, a NSC-specific transcription factor. (f) FACS analysis of TRA-1-81 in WT and *PTEN*$^{-/-}$ NSCs, which shows the absence of TRA-81 in NSCs, a pluripotency-associated antigen. (g) RT-qPCR showing the expression of GFAP, an astrocyte marker, in NSC spontaneously differentiated derivatives, but not in NSCs, MSCs, and fibroblasts. n=3.

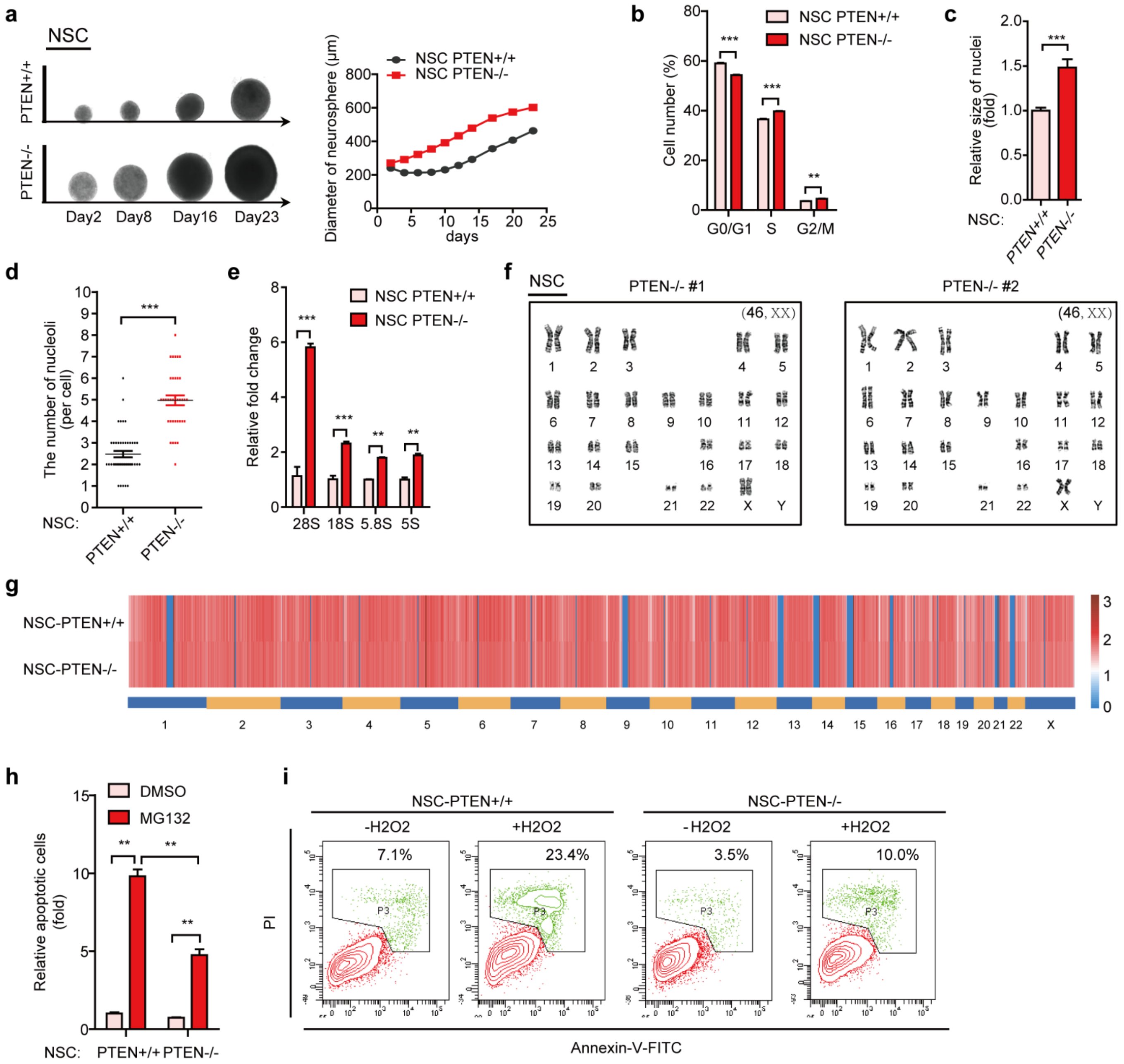


**Supplementary Figure 3. Phenotypic characterization in PTEN-deficient NSCs *in vitro*.** (a) *PTEN*$^{-/-}$ NSCs cultured as neurospheres showed accelerated cell proliferation, compared with WT NSCs. Diameters of neurospheres were quantified. n=8. (b) Cell cycle analysis of *PTEN*$^{-/-}$ and WT NSCs. n=3. **, $p$<0.01 (t-test); and ***, $p$<0.001 (t-test). (c) The size (area) of nuclei in NSCs was determined by image J. n=100. ***, $p$<0.001 (t-test). (d) Immunostaining of nucleolin showing increased nucleolus number in *PTEN*$^{-/-}$ NSCs, relative to WT NSCs. n=100. ***, $p$<0.001 (t-test). (e) RT-qPCR analysis of rRNA transcripts in WT and *PTEN*$^{-/-}$ NSCs. n=3. **, $p$<0.01 (t-test); and ***, $p$<0.001 (t-test). (f) Karyotyping analysis of PTEN-deficient NSC lines showed no chromosomal aberrance after extensive *in vitro* passaging (passage 12). (g) Genome-wide CNV analysis of WT and *PTEN*$^{-/-}$ NSCs by deep sequencing. No significant CNV alteration between WT and *PTEN*$^{-/-}$ NSCs was observed. (h) FACS analysis of cell apoptosis in MG132-treated WT and *PTEN*$^{-/-}$ NSCs. n=3. **, $p$<0.01 (t-test). (i) FACS analysis of cell apoptosis in $H_2O_2$-treated WT and *PTEN*$^{-/-}$ NSCs.

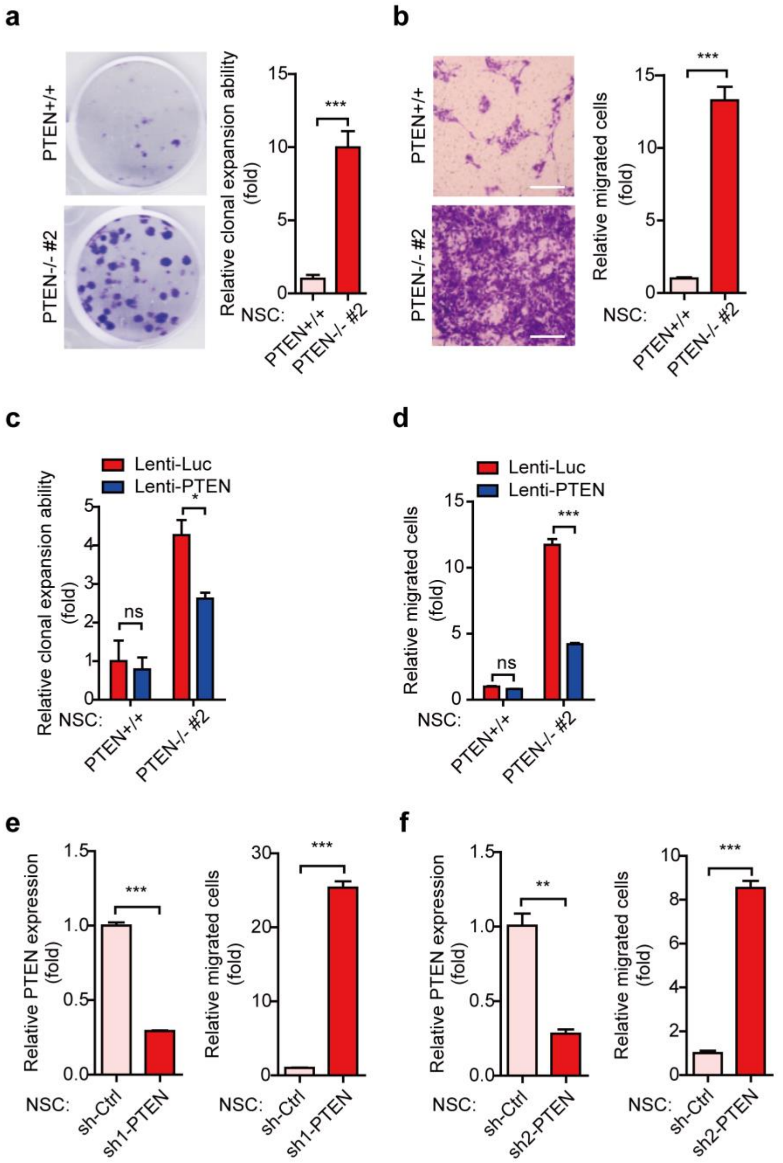


**Supplementary Figure 4. Phenotypic analysis in PTEN-deficient NSCs *in vitro*.** (a) Clonal expansion analysis in WT and *PTEN*$^{-/-}$ NSCs (line #2). Crystal violet staining-positive cells were calculated and presented as fold induction using Image J. n=4. ***, *p*<0.001 (t-test). (b) Cell migration analysis of WT and *PTEN*$^{-/-}$ NSCs (line #2). Relative cell migration efficiency was determined. n=6. ***, *p*<0.001 (t-test). Scale bars, 1 mm. (c and d) Clonal expansion (c) and cell migration (d) analyses of WT and *PTEN*$^{-/-}$ NSCs (line #2) transduced with a lentiviral PTEN expression vector or a control vector (Luc). n=3 (c) and n=6 (d). *, *p*<0.05 (t-test); ***, *p*<0.001 (t-test); and ns, not significant. (e and f) Migration ability analysis of WT NSCs transduced with PTEN-specific shRNA #1 (e) and shRNA #2 (f) or Ctrl shRNA. Relative cell migration efficiency was determined. n=6. **, *p*<0.01 (t-test); and ***, *p*<0.001 (t-test).

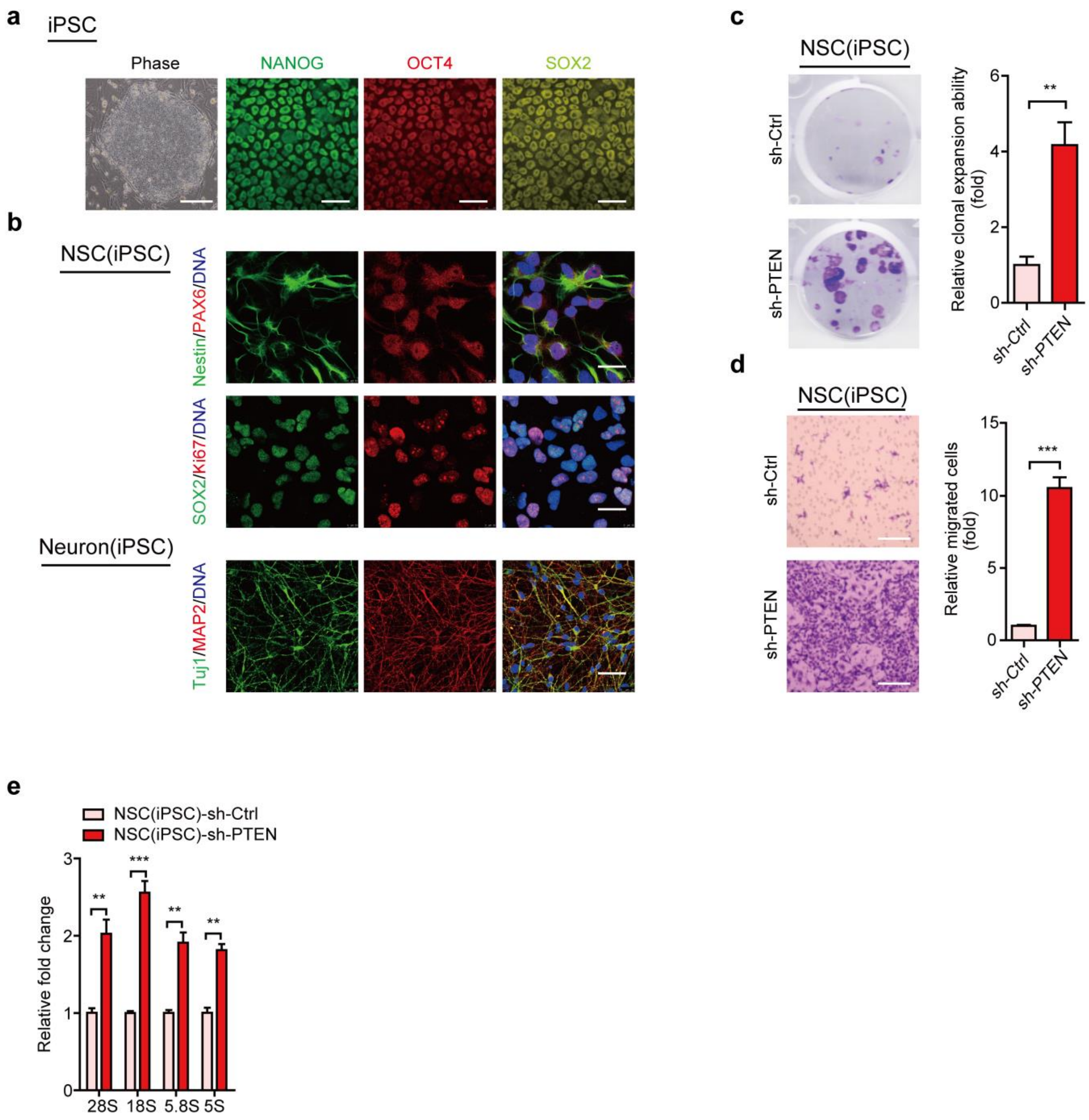


**Supplementary Figure 5. Phenotypic analysis in human iPSC-derived NSCs.** (a) Immunofluorescence analysis of the pluripotency markers in iPSCs derived from human dermal fibroblasts. Scale bars, 1 mm (phase) and 50 μm (IF). (b) Immunostaining of neural progenitor (upper panels) and neuron (lower panels)-specific markers in iPSCs-derived NSCs (upper panels) and their neuronal derivatives (lower panels). Nuclei were stained with Hoechst 33342. Scale bars, 25 μm (NSCs) and 50 μm (neurons). (c) Clonal expansion analysis in human iPSC-derived NSCs expressing control or PTEN shRNA . n=4. **, $p<0.01$ (t-test). (d) Cell migration analysis in human iPSC-derived NSCs expressing control or PTEN shRNA . n=6. ***, $p<0.001$ (t-test). Scale bars, 1 mm. (e) RT-qPCR analysis of rRNA transcripts in human iPSCs derived NSCs expressing control or PTEN shRNA. n=3. **, $p<0.01$ (t-test); and ***, $p<0.001$ (t-test).

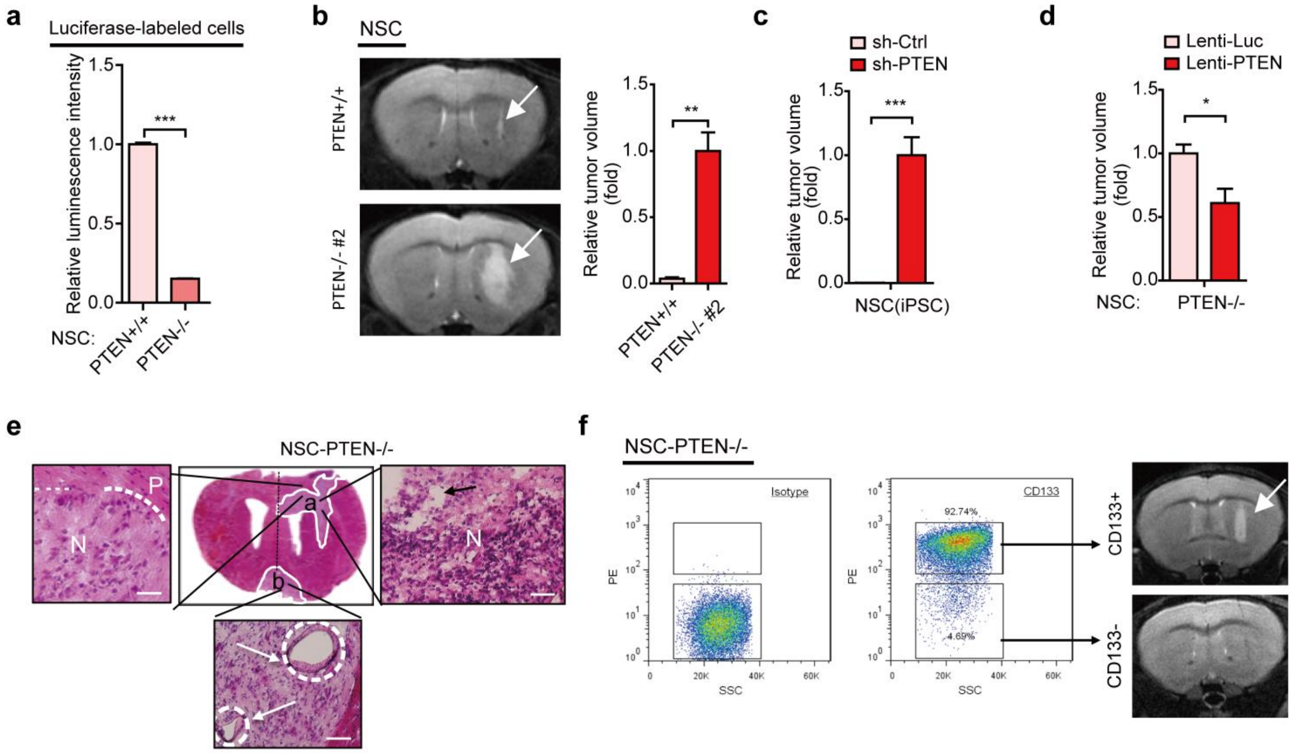


**Supplementary Figure 6. Phenotypic analysis in PTEN-deficient NSCs *in vivo*.** (a) Luminescence analysis of luciferase activity in WT and *PTEN*$^{-/-}$ NSCs transduced with lenti-Luciferase just before implantation (also see figure 2e). The results indicated that the activity of luciferase in *PTEN*$^{-/-}$ NSCs was even lower than in WT NSCs. (b) MRI analyses of intracranially implanted WT and *PTEN*$^{-/-}$ NSCs (line #2). n=3. **, p<0.01 (t-test). (c) MRI analyses of intracranially implanted human iPSC-derived NSCs pre-transduced with control or PTEN shRNA. Relative tumor volumes are presented. n=7. ***, p<0.001 (t-test). (d) MRI analyses of intracranially implanted *PTEN*$^{-/-}$ NSCs (line #1) pre-transduced with a PTEN expression vector or a control vector (Luc). Relative tumor volumes are presented. n=7 for lenti-Luc group and n=6 for lenti-PTEN group. *, p<0.05 (t-test). (e) H&E staining analysis of brain sections, 70 days after being implanted with *PTEN*$^{-/-}$ NSCs into the right hemisphere. a, regions around the injected site; b, a region comprised of migrated neoplastic cells. N represents neoplasm, and P represents mouse brain parenchyma. Occasionally we were able to observe intra-tumor vasculatures (white arrows) and non-classic necrosis regions (black arrow). Scale bars, 500 μm. (f) MRI analysis of the brain of NOD/SCID mice receiving implantation with CD133-positive (upper) and CD133-negative (below) cells sorted from *PTEN*$^{-/-}$ NSCs. Representative data from 3 independent experiments are shown.

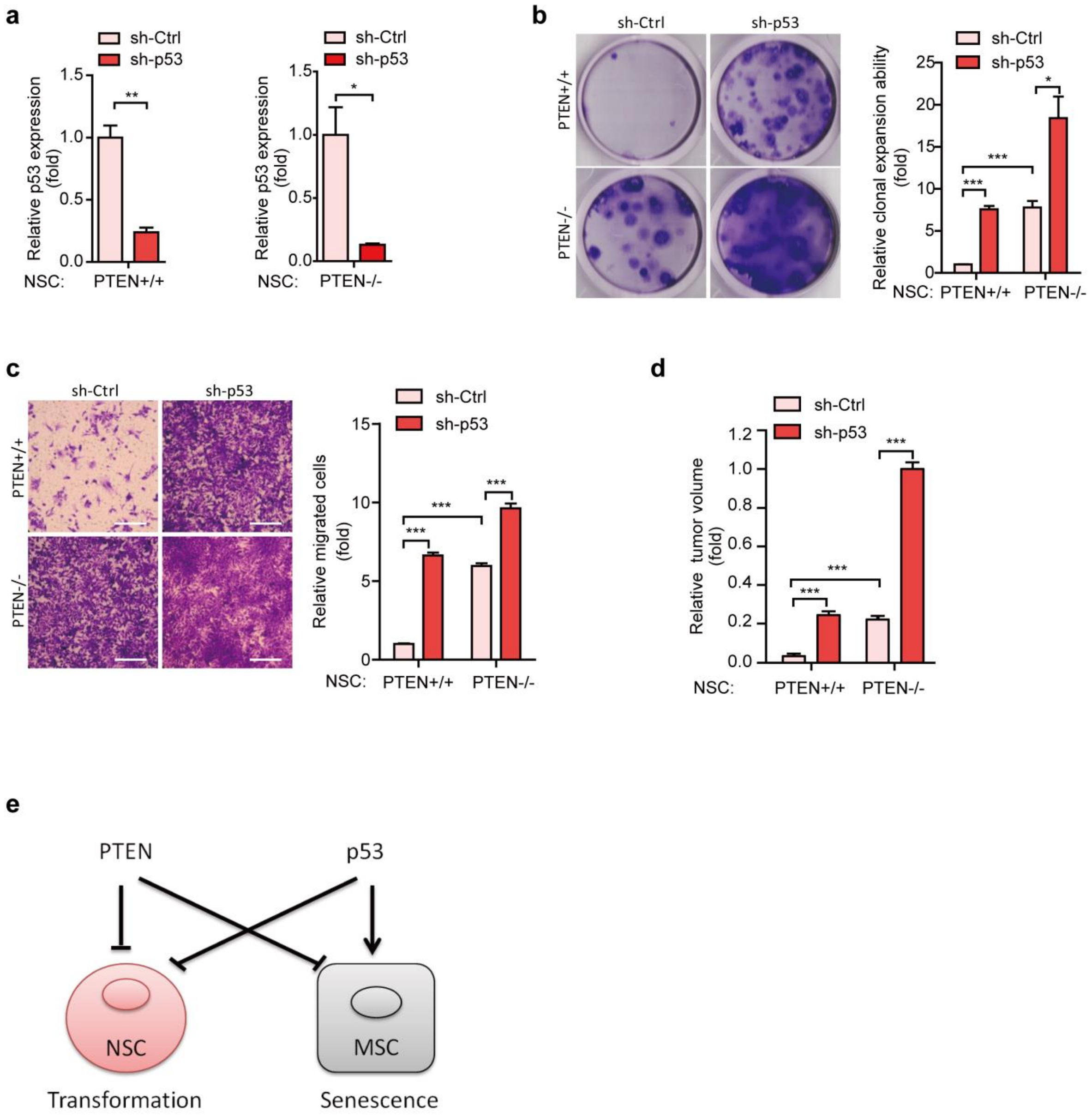


**Supplementary Figure 7. Phenotypic analyses in p53- and PTEN-deficient NSCs.** (a) Knockdown of endogenous p53 in *PTEN*$^{+/+}$ NSCs (left) and *PTEN*$^{-/-}$ NSCs (right) by a lentiviral vector encoding a p53-specific shRNA. (b and c) A cooperative effect of p53 knockdown and PTEN knockout in enhancing the clonal expansion (b) and migration (c) abilities of NSCs. (d) A cooperative role of PTEN knockout and p53 knockdown in promoting NSC neoplastic transformation in mouse brain. *PTEN*$^{+/+}$ NSCs and *PTEN*$^{-/-}$ NSCs were transduced with a p53 shRNA expression vector or a control vector. Relative tumor volumes were quantified by measuring the numbers of the GFP (expressed constitutively from shRNA vector) positive engrafted cells. n=4, ***, p<0.001 (t-test); and ns, not significant. (e) A putative model depicting different roles of PTEN and p53 in determining the fates of human NSCs and MSCs.

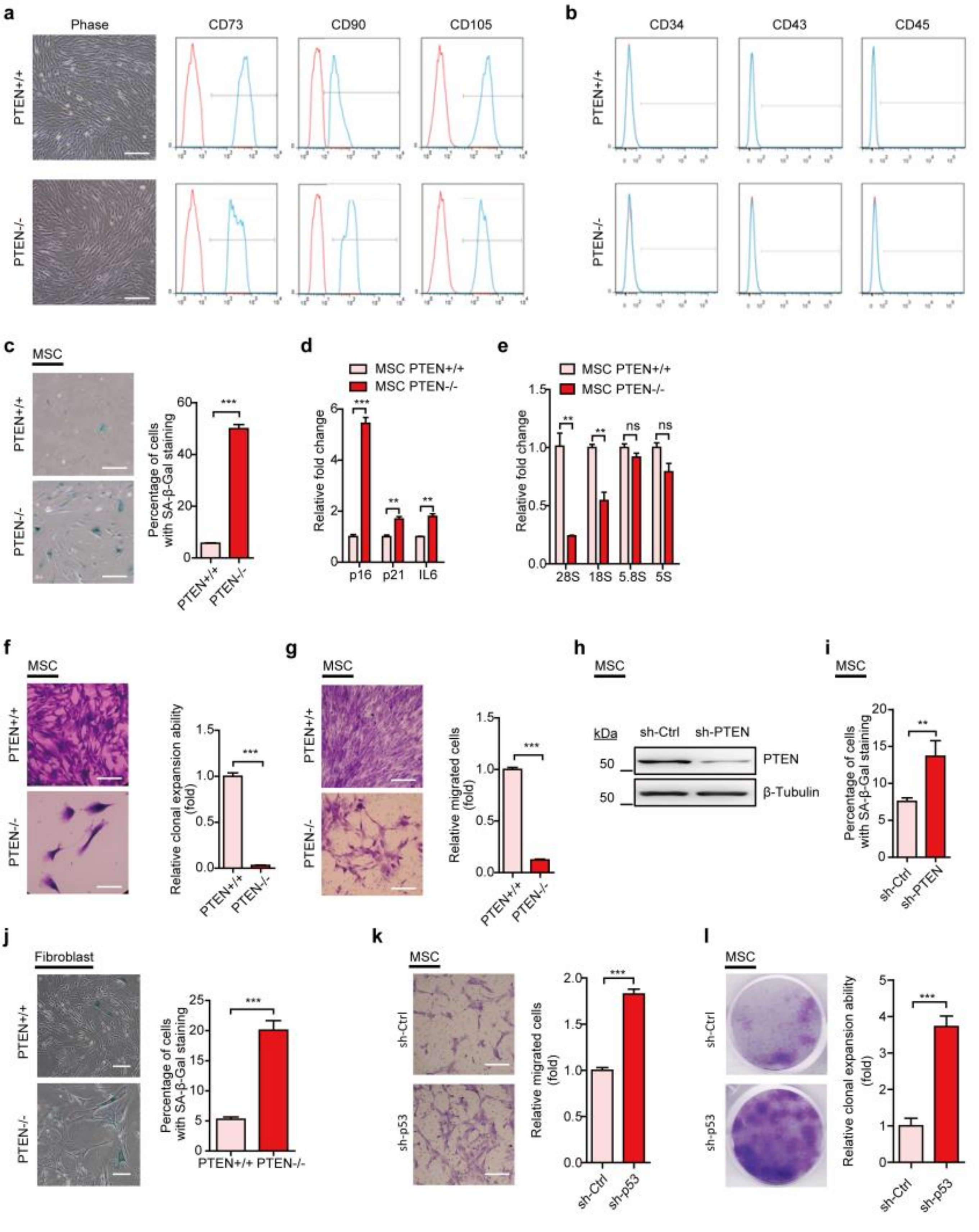


**Supplementary Figure 8. PTEN-deficient MSCs showed characteristic features of premature cellular senescence.** (a) FACS analysis of mesenchymal stem cell-specific surface markers (CD73, CD90, and CD105) in MSCs differentiated from WT and *PTEN*$^{-/-}$ ESCs. Scale bars, 1 mm. (b) Absence of expression of CD34, CD43, and CD45 in MSCs differentiated from WT and *PTEN*$^{-/-}$ ESCs, determined by FACS. (c) Senescence-associated (SA)-β-gal staining in WT and *PTEN*$^{-/-}$ MSCs at passage 2. n=3. ***, $p$<0.001 (t-test). Scale bars, 1 mm. (d-e)

RT-qPCR analysis of senescence-associated genes (d) and rRNA transcripts (e) in WT and *PTEN*$^{-/-}$ MSCs. n=3. **, *p*<0.01 (t-test); ***, *p*<0.001 (t-test); ns, not significant. (f) Compromised clonal expansion ability in *PTEN*$^{-/-}$ MSCs. Relative expanded cells were visualized via crystal violet staining. n=3, ***, *p*<0.001 (t-test). Scale bars, 1 mm. (g) Compromised migration ability in *PTEN*$^{-/-}$ MSCs. n=3, ***, *p*<0.001 (t-test). Scale bars, 1 mm. (h and i) Knockdown of PTEN in human primary bone marrow-derived mesenchymal stem cells (BM-MSCs) resulted in premature cellular senescence. Immunoblots indicated diminished PTEN levels in MSCs transduced with lentiviral PTEN shRNA vector. β-Tubulin was used as loading control (h). The senescence-associated (SA)-β-gal staining showed induction of cellular senescence upon PTEN deficiency (i). n=3. **, *p*<0.01 (t-test). (j) The SA-β-gal staining showed increased cellular senescence in PTEN-deficient fibroblast-like cells derived from *in vivo* differentiated *PTEN*$^{-/-}$ ESCs. n=3. ***, *p*<0.001 (t-test). (k) Knockdown of p53 in human primary BM-MSCs led to increased migration ability. Cell migration abilities of MSCs transduced with lentiviral vector encoding a p53-specific shRNA or a control-shRNA are shown. n=6. ***, *p*<0.001 (t-test). Scale bars, 1 mm. (l) Knockdown of p53 in human primary BM-MSCs led to strengthened cellular proliferation. Clonal expansion abilities of MSCs transduced with lentiviral vector encoding a p53-specific shRNA or a control-shRNA are shown. n=4. ***, *p*<0.001 (t-test).

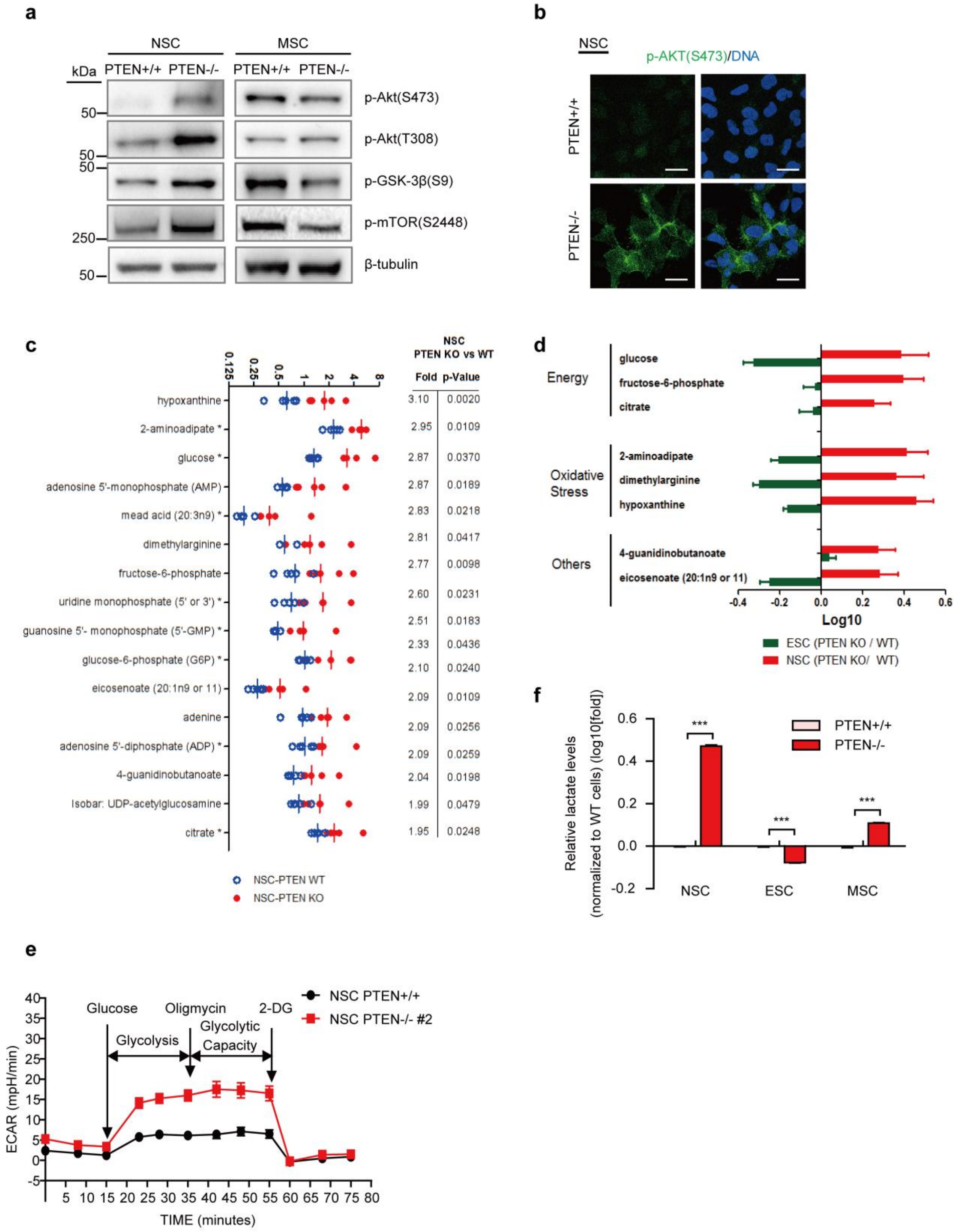


**Supplementary Figure 9. PTEN-deficient NSCs displayed metabolic changes favoring oncogenic identity.** (a) Immunoblotting analyses with indicated antibodies in WT and PTEN-deficient NSCs and MSCs. (b) Immunofluorescence analysis of phospho-AKT in WT and *PTEN*$^{-/-}$ NSCs. (c) Metabolomics analysis showed differential metabolite profiles between WT and *PTEN*$^{-/-}$ NSCs. n=6. (d) Elevation of oncogenic metabolites in *PTEN*$^{-/-}$ NSCs, but not in their ESC counterparts. n=6. (e) Glycolysis flux analysis measured by ECARs in WT and *PTEN*$^{-/-}$ NSCs (line #2). n=5. (f) Lactate levels in WT and PTEN-deficient NSCs, MSCs, and ESCs. n=4. ***, $p$ <0.001 (t-test).

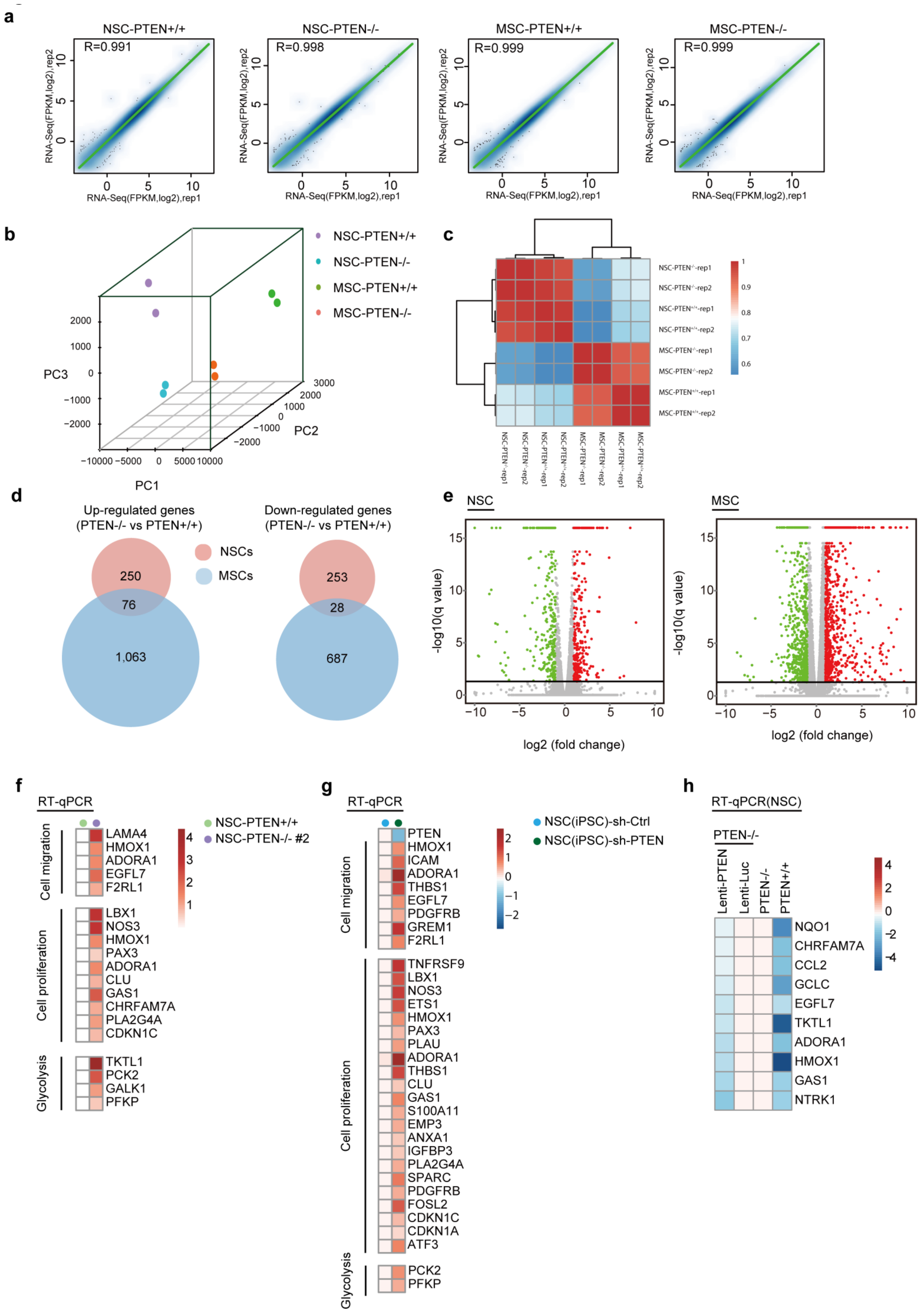


**Supplementary Figure 10. Gene expression profile in WT and PTEN-deficient stem cells.** (a) Scatter plots

showing the correlation of gene expression (FPKM > 0.1) between duplicates of WT and *PTEN*$^{-/-}$ NSCs and MSCs, respectively. The Pearson correlation coefficient was shown at the left upper side of each panel. (b) Principal component analysis (PCA) of RNA-Seq data of WT and *PTEN*$^{-/-}$ NSCs and MSCs using genes of FPKM > 0.1. (c) Heatmap showing correlation of gene expression between 8 samples. (d) Venn diagrams showing the overlap between the up-regulated (q-value < 0.05 FC [*PTEN*$^{-/-}$/*PTEN*$^{+/+}$] > 2) or down-regulated (q-value < 0.05, FC [*PTEN*$^{-/-}$/*PTEN*$^{+/+}$] < 0.5) genes in NSCs and MSCs, respectively. (e) Volcano plots show gene expression changes between *PTEN*$^{-/-}$ and WT in NSCs (left) and MSCs (right). Genes up-regulated (q-value < 0.05, FC[*PTEN*$^{-/-}$/*PTEN*$^{+/+}$] > 2) after PTEN knockout were depicted in red dots and those down-regulated （q-value < 0.05, FC[PTEN$^{-/-}$/PTEN$^{+/+}$] < 0.5） were in green dots. (f and g) Heatmap of RT-qPCR results showing marked upregulation of tumor-related genes in *PTEN*$^{-/-}$ #2 NSCs (f) and iPSC-derived NSCs transduced with lenti-sh-*PTEN* (g). (h) Heatmap of RT-qPCR results showing that re-introduction of PTEN in *PTEN*$^{-/-}$ NSCs could partially decrease the expression of the indicated tumor-related genes.

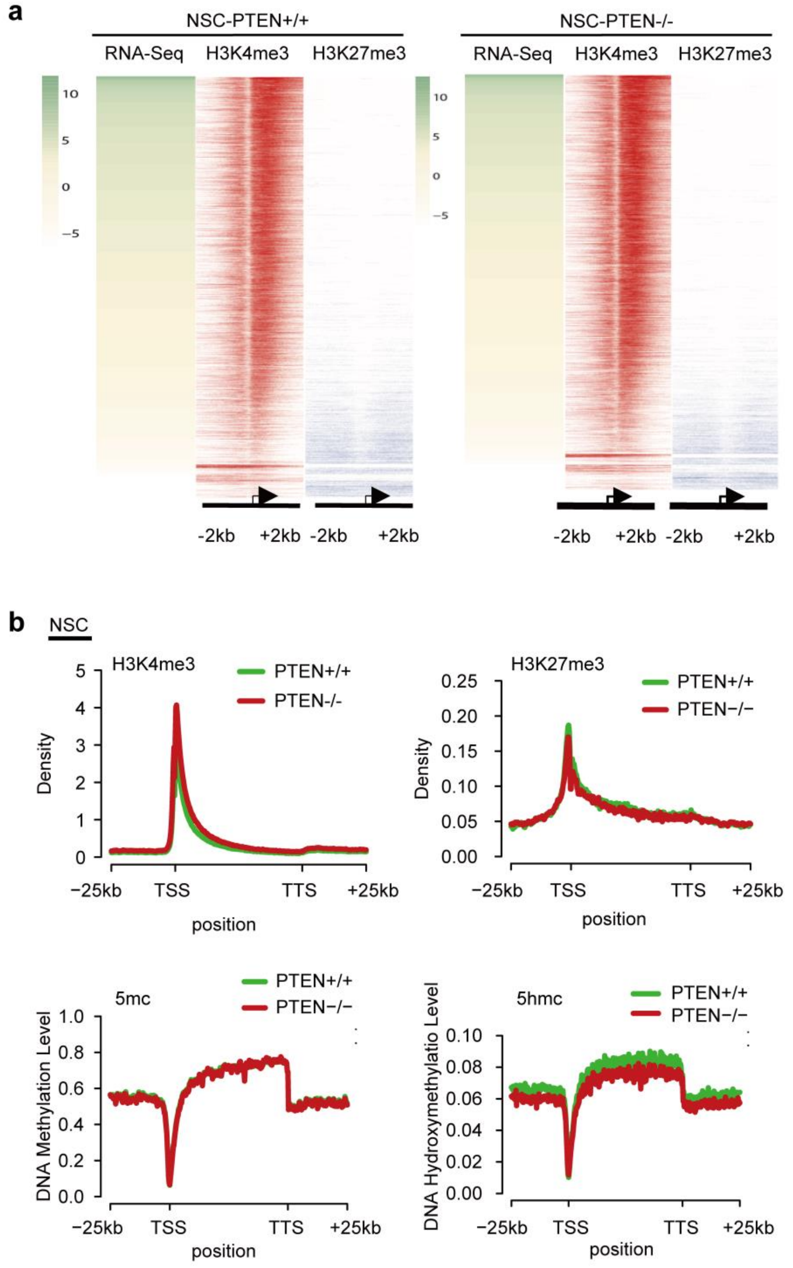


**Supplementary Figure 11. Global histone and DNA modifications in WT and PTEN-deficient NSCs.** (a) Heatmap showing enrichment of H3K4me3 and H3K27me3 sorted by RNA-signal at genomic regions between -2 kb and +2 kb around transcription start sites (TSSs). (b) The distribution of epigenetic modifications H3K4me3, H3K27me3, 5mc, and 5hmc around the gene body regions in WT and *PTEN*$^{-/-}$ NSCs.

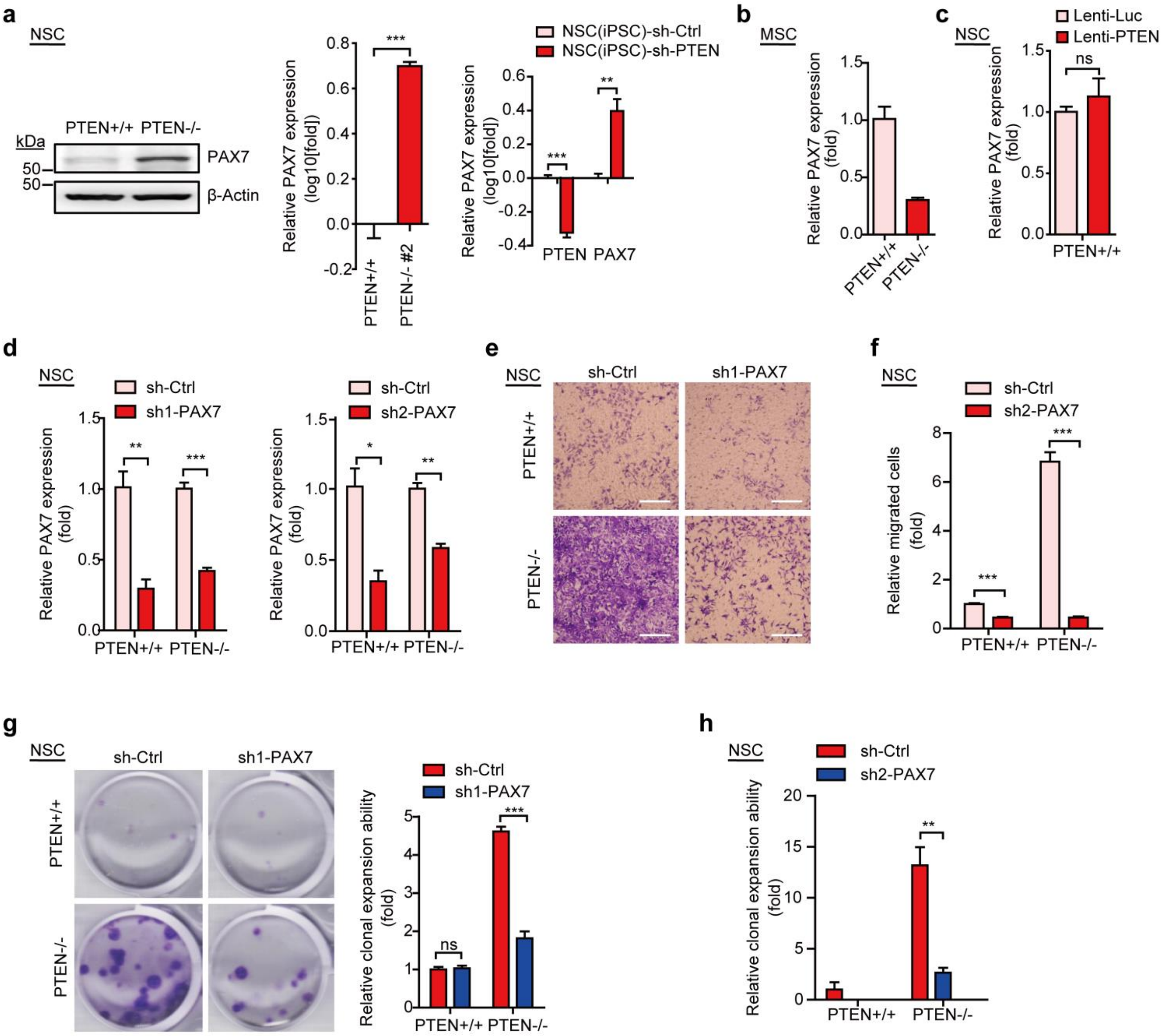


**Supplementary Figure 12. Elevated levels of PAX7 underlie neoplastic phenotypes in PTEN-deficient NSCs.** (a) Immunoblotting analyses of PAX7 expression in WT and *PTEN*$^{-/-}$ NSCs (line#1, left). β-Actin was used as loading control. RT-qPCR analysis of *PAX7* mRNA in WT and *PTEN*$^{-/-}$ NSCs (line#2, middle) and in human iPSC-derived NSCs expressing control or PTEN shRNA (right). n=3. ***, $p$<0.001 (t-test); ***, $p$<0.001 (t-test). (b) RT-qPCR analysis of *PAX7* mRNA in WT and *PTEN*$^{-/-}$ MSCs. n=3. (c) RT-qPCR analysis of *PAX7* mRNA in WT NSCs transduced with lentivirus encoding PTEN. n=3. ns, not significant. (d) RT-qPCR analysis showing downregulation of PAX7 expression in WT and *PTEN*$^{-/-}$ NSCs transduced with control shRNA or PAX7 shRNA lentiviral vector (sh1 and sh2 are two independent PTEN shRNAs). n=3. *, $p$<0.05 (t-test); **, $p$<0.01 (t-test); and ***, $p$<0.001 (t-test). (e) Representative images of migrated cells in WT and *PTEN*$^{-/-}$ NSCs transduced with control shRNA or PAX7 shRNA #1 (also see Fig. 4c.). Scale bars, 1 mm. (f) Cell migration analyses in WT and *PTEN*$^{-/-}$ NSCs transduced with control shRNA or PAX7 shRNA #2. n=3. ***, $p$<0.001 (t-test). (g) Clonal expansion analyses in WT and *PTEN*$^{-/-}$ NSCs transduced with control shRNA or PAX7 shRNA #1. n=3. ***, $p$<0.001 (t-test); and ns, not significant. (h) Clonal expansion analyses in WT and *PTEN*$^{-/-}$ NSCs transduced with control shRNA or PAX7 shRNA #2. n=3. **, $p$<0.01 (t-test).

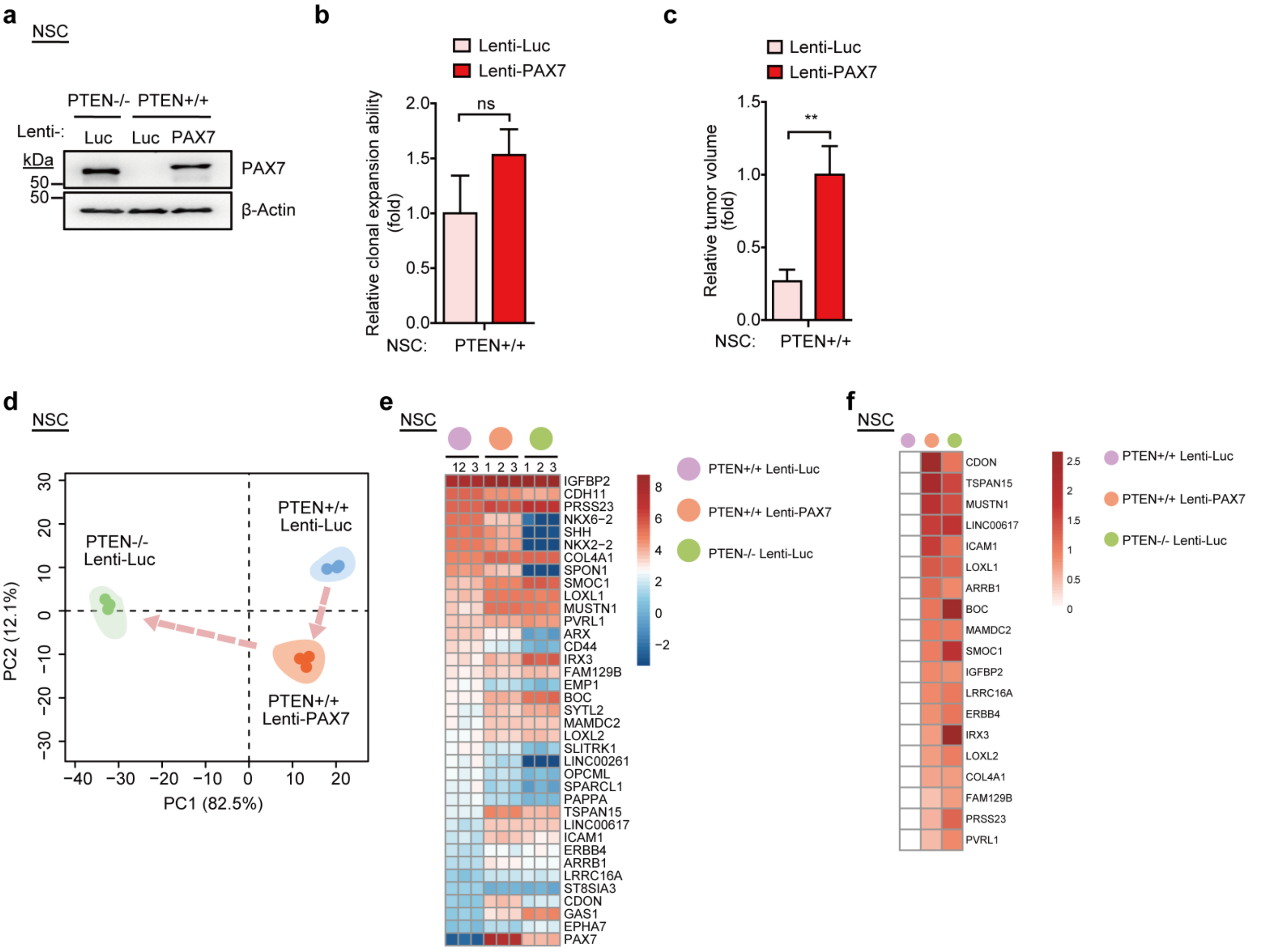


**Supplementary Figure 13. Overexpression of PAX7 in WT NSCs partially recapitulated the neoplastic phenotypes in PTEN-deficient NSCs.** (a) Immunoblotting analysis of PAX7 expressions in *PTEN*$^{-/-}$ NSCs and WT NSCs transduced with the PAX7 expression vector. β-Actin was used as loading control. (b) *In vitro* clonal expansion analyses in WT NSCs transduced with the PAX7 expression vector or luciferase control vector. n=3. ns, not significant. (c) MRI analysis of tumor volumes in mouse brains implanted with WT NSCs pre-transduced with the PAX7 expression vector or luciferase control vector. Relative tumor volumes are presented. n=5. **, $p$<0.01 (t-test). (d) PCA analysis of *PTEN*$^{+/+}$ Lenti-Luc, *PTEN*$^{+/+}$ Lenti-PAX7 and *PTEN*$^{-/-}$ Lenti-Luc. PAX7 overexpression in *PTEN*$^{+/+}$ Lenti-Luc moved toward *PTEN*$^{-/-}$ Lenti-Luc on the PC1 and PC2 directions. (e) Heatmap showing log2 (FPKM) of 37 genes which were up-regulated or down-regulated in the *PTEN*$^{-/-}$ Lenti-Luc vs *PTEN*$^{+/+}$ Lenti-Luc and *PTEN*$^{+/+}$ Lenti-PAX7 vs *PTEN*$^{+/+}$ Lenti-Luc at the same time. (f) 19 genes out of 37 genes which were related to tumorigenesis. *PTEN*$^{+/+}$ Lenti-PAX7 and *PTEN*$^{-/-}$ Lenti-Luc were normalized by *PTEN*$^{+/+}$ Lenti-Luc (value=1).

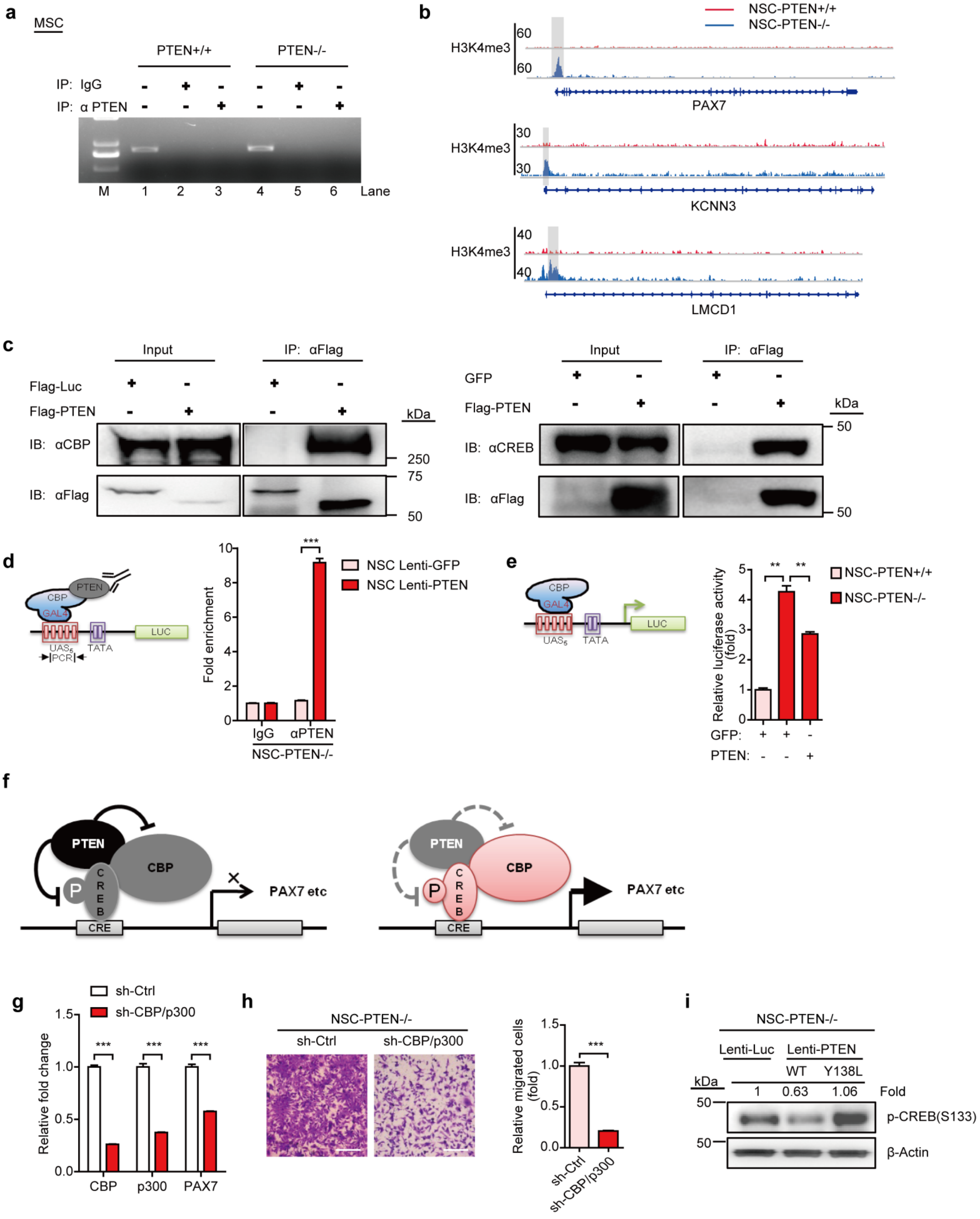


**Supplementary Figure 14. Interplay between PTEN and CREB/CBP underlie neoplastic features in PTEN-deficient NSCs.** (a) ChIP-PCR showing no association of endogenous PTEN with *PAX7* promoter in WT MSCs. The *PAX7* promoter was amplified by PCR from either genomic DNA as input (lanes 1 and 4) or anti-PTEN immunoprecipitated DNA (lanes 3 and 6) (also see Fig. 4g for NSC data). (b) H3K4me3 signal tracks in WT and *PTEN*$^{-/-}$ NSCs for the loci of PAX7, KCNN3, and LMCD1, three representative CREB-target genes. (c)

Co-immunoprecipitation (Co-IP) of endogenous CBP (left blots) and CREB (right blots) with exogenously expressed flag-tagged PTEN in HEK293T cells; Flag-Luciferase (left) and GFP (right) were used as controls. (d) PTEN was recruited to the gene promoter by CBP. ChIP analysis of the PTEN recruited to promoter-tethered CBP in *PTEN*$^{-/-}$ NSCs transfected with $(UAS)_5$-TATA-luc reporter, together with or without PTEN. PCR was used to amplify a UAS-containing DNA fragment. n=6, ***, $p<0.001$ (t-test). (e) Effect of PTEN on CBP transactivation ability in NSCs. WT and *PTEN*$^{-/-}$ NSCs were transfected with GAL4-CBP, (UAS)5-TATA-Luc-pG5, together with or without PTEN expression vector. Relative luciferase activity was determined as fold induction. n=3, **, $p<0.01$ (t-test). (f) A putative model of PTEN/CREB/CBP interactions in the regulation of *PAX7* gene expression in NSCs. (g) mRNA levels of CBP, p300, and PAX7 in NSCs transduced with a CBP/p300-specific shRNA. n=3. ***, $p<0.001$ (t-test). (h) Cell migration analysis of *PTEN*$^{-/-}$ NSCs transduced with the lentiviral vector encoding CBP/p300-specific shRNA (sh-CBP/p300) or a control vector (sh-Ctrl). Scale bars, 1 mm. n=3. ***, $p<0.001$ (t-test). (i) Immunoblotting analyses of phospho-CREB in *PTEN*$^{-/-}$ NSCs transduced with the lentiviral vector encoding WT- or protein phosphatase-dead Y138L mutant PTEN or a luciferase control (Luc). β-Actin was used as loading control.

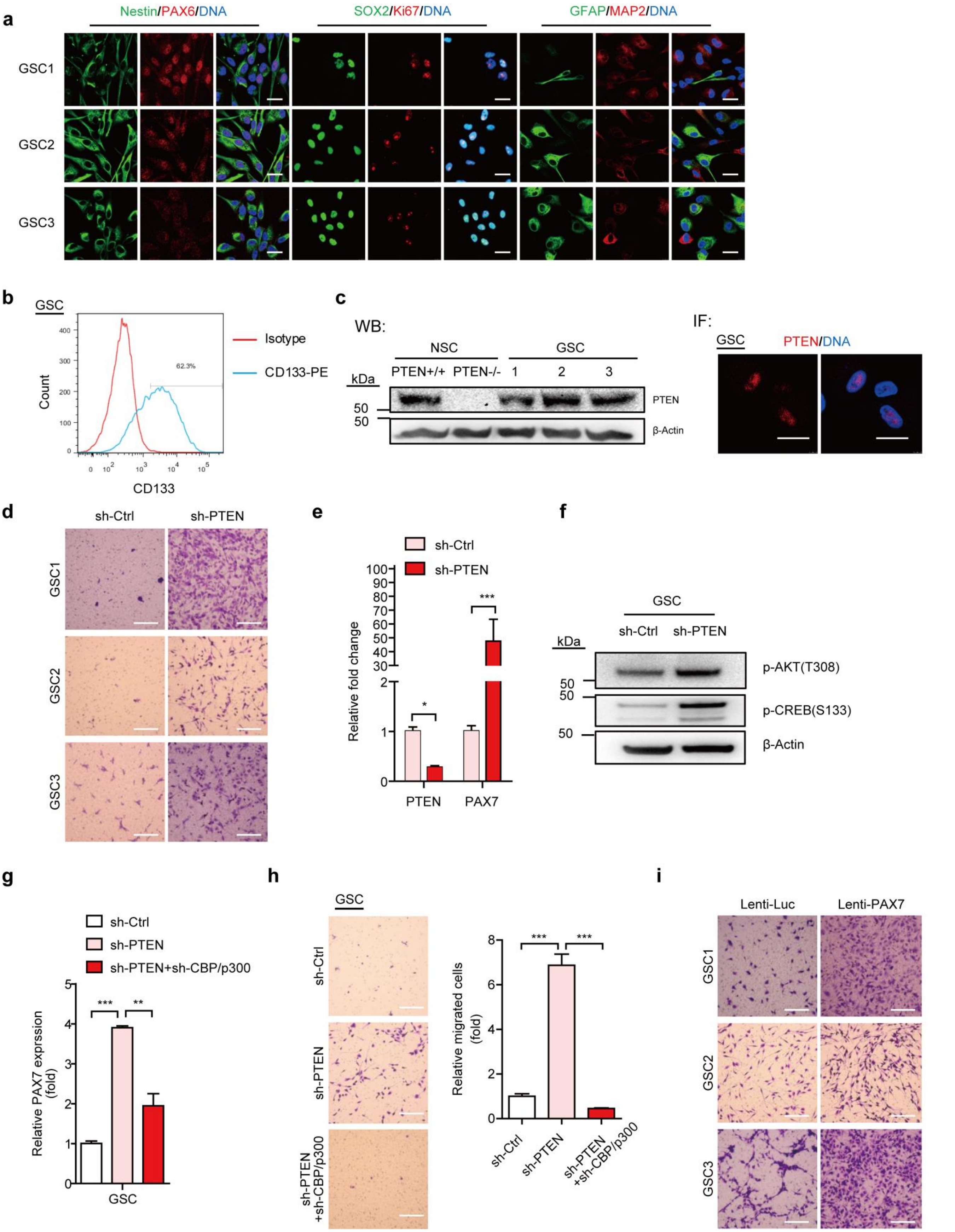


**Supplementary Figure 15. Phenotypic analyses of GSCs.** (a) Immunofluorescence analysis of GSC-specific markers in three lines of GSCs (GSC1, GSC2, GSC3, respectively). Nuclei were stained with Hoechst 33342. Scale bars, 25 μm. (b) Representative FACS analysis of CD133 in GSCs. (c) Left panel: Immunobloting analysis of PTEN in WT and *PTEN*$^{-/-}$ NSCs, as well as three lines of GSCs. Right panel: Immunofluorescence showing

PTEN is expressed in the nuclei of GSCs. Scale bars, 25 μm. (d) Representative cell migration assays showing PTEN-knockdown stimulated the migration ability of GSCs (also see Fig. 5c). Scale bars, 1 mm. (e) RT-qPCR showing that knockdown of PTEN in GSCs (line #3) upregulated expressions of *PAX7*. n=3 *, $p<0.05$ (t-test); ***, $p<0.001$ (t-test). (f) Immunobloting analysis of phospho-AKT (T308) and phospho-CREB (S133) in GSCs (line #3) transduced with a control shRNA or PTEN shRNA. β-Actin was used as loading control. (g) RT-qPCR analysis of *PAX7* mRNA in GSCs (line #3) transduced with control shRNA or PTEN shRNA or PTEN shRNA plus CBP/p300 shRNA. n=3. **, $p<0.01$ (t-test); ***, $p<0.001$ (t-test). (h) Cell migration analyses in GSCs (line #3) transduced with control shRNA or PTEN shRNA or PTEN shRNA plus CBP/p300 shRNA. n=6. ***, $p<0.001$ (t-test). (i) Representative migration assays showing that PAX7 overexpression enhanced the migration ability of GSCs (also see Fig. 5d). Scale bars, 1mm.

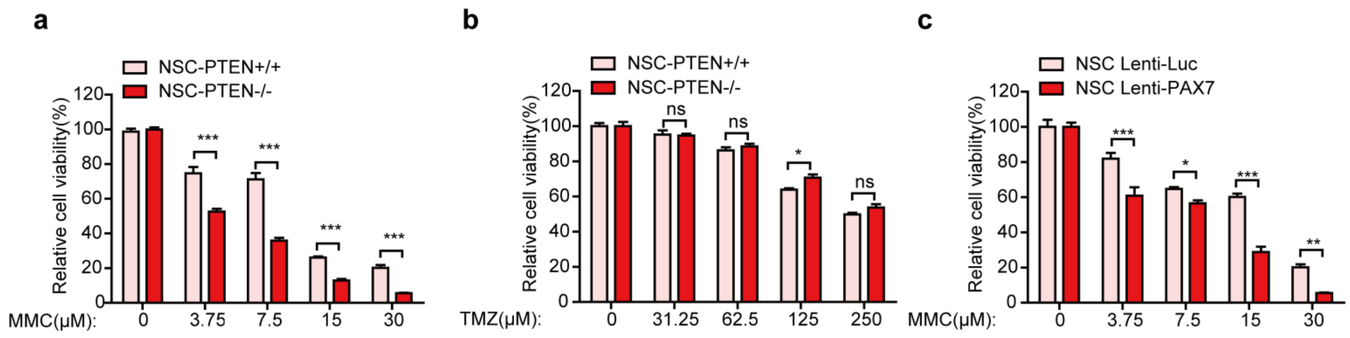


**Supplementary Figure 16. MMC preferentially killed PTEN-deficient or PAX7-overexpressed NSCs.** (a and b) Cell viability analysis in MMC (24 h) (a) or TMZ (48 h) (b)-treated WT and *PTEN*$^{-/-}$ NSCs. n=6, *, $p$<0.05 (t-test); ***, $p$<0.001 (t-test); ns, not significant. (c) Cell viability analysis in MMC (24 h)-treated NSCs with PAX7 overexpression. n=6, *, $p$<0.05 (t-test); **, $p$<0.01 (t-test); ***, $p$<0.001 (t-test).

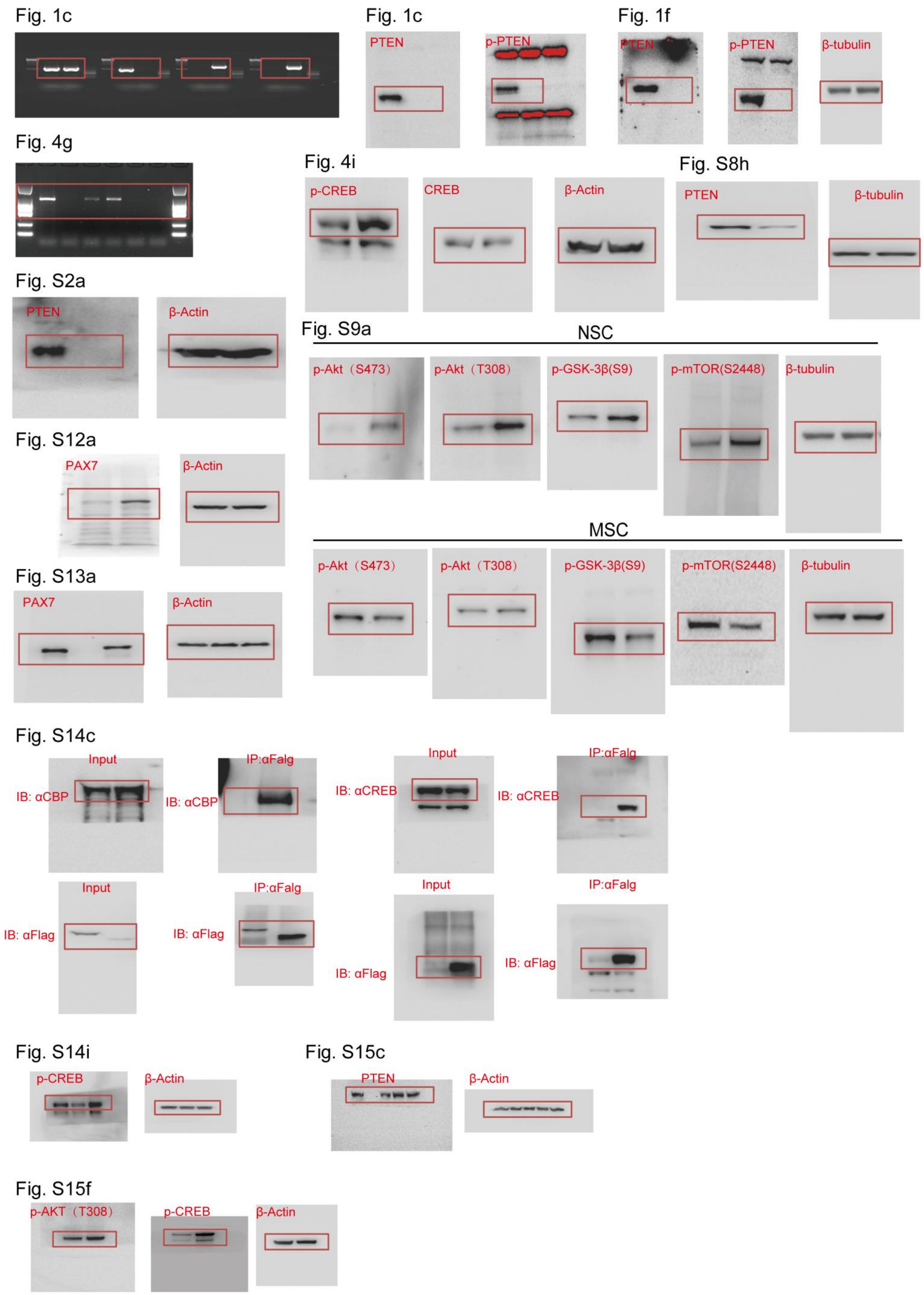


**Supplementary Figure 17. Uncropped scans of Gels and Western blots.**

**Supplementary Table 1. Characterization of GSC lines**

| Cell Line | Age[a] | Sex | %CD133$^{+}$ in line | Nestin | PAX6 | SOX2 | GFAP | MAP2 | Tuj1 |
|---|---|---|---|---|---|---|---|---|---|
| GSC1 | 68 | M | 62.3 | + | + | + | + | + | + |
| GSC2 | 46 | M | 17.2 | + | + | + | + | + | + |
| GSC3 | 63 | M | 32.9 | + | + | + | + | + | + |

Abbreviations: M, male; [a] Age when operation is performed

**Supplementary Table 2. Compounds used in this study**

| Compound name | Concentrations used (μM) | References |
|---|---|---|
| WP1066 | 0,2.5,5,10,20 | [1, 2] |
| PX12 | 0,62.5,125 | [3] |
| Sulforaphane | 0,62.5,125, 250,500 | [4, 5] |
| Temsirolimas | 0,12.5,25,50,100 | [6, 7] |
| Narciclasine | 0,1.25,2.5,5,10 | [8, 9] |
| Temozolomide | 0,31.25,62.5,125,250 | [10, 11, 12] |
| Olaparib | 0,12.5,25,50,100 | [13, 14] |
| Campathecin | 0,1.25,2.5,5,10 | [15, 16] |
| Hydroxyurea | 0,62.5,125,250,500 | [17] |
| Mitomycin C | 0.3.75,7.5,15,30 | [18, 19] |

**Supplementary Table3. Primer list**

| **Primer** | **Sequence (5' to 3')** | **Application** |
|---|---|---|
| h-CBP-F | GCAACCCCAAAAGAGCCAAACT | qPCR |
| h-CBP-R | CCTCGTAGAAGCTCCGACAGT | qPCR |
| h-p300-F | AGCCAAGCGGCCTAAACTC | qPCR |
| h-p300-R | TCACCACCATTGGTTAGTCCC | qPCR |
| h-NQO1-F | CTCCGCAAACACAAGACGAAC | qPCR |
| h-NQO1-R | CACATGGGCCGTGTAGAGTC | qPCR |
| h-β-Actin-F | CATGTACGTTGCTATCCAGGC | qPCR |
| h-β-Actin-R | CTCCTTAATGTCACGCACGAT | qPCR |
| h-PTEN-F | GTTTACCGGCAGCATCAAAT | qPCR |
| h-PTEN-R | CCCCCACTTTAGTGCACAGT | qPCR |
| h-CCL2-F | CAGCCAGATGCAATCAATGCC | qPCR |
| h-CCL2-R | TGGAATCCTGAACCCACTTCT | qPCR |
| h-HMOX1(HO1)-F | AAGACTGCGTTCCTGCTCAAC | qPCR |
| h-HMOX1(HO1)-R | AAAGCCCTACAGCAACTGTCG | qPCR |
| h-GCLC-F | GGAGACCAGAGTATGGGAGTT | qPCR |
| h-GCLC-R | CCGGCGTTTTCGCATGTTG | qPCR |
| h-PAX7-F | ACCCCTGCCTAACCACATC | qPCR |
| h-PAX7-R | GCGGCAAAGAATCTTGGAGAC | qPCR |
| h-NOS3-F | TGATGGCGAAGCGAGTGAAG | qPCR |
| h-NOS3-R | ACTCATCCATACACAGGACCC | qPCR |
| h-THBS1-F | AGACTCCGCATCGCAAAGG | qPCR |
| h-THBS1-R | TCACCACGTTGTTGTCAAGGG | qPCR |
| h-ADORA1-F | CCACAGACCTACTTCCACACC | qPCR |
| h-ADORA1-R | TACCGGAGAGGGATCTTGACC | qPCR |
| h-NTRK1-F | GCTGGCTCTTCAATGGCTC | qPCR |
| h-NTRK1-R | GTGTAGTTGCCGTTGTTGACG | qPCR |
| h-GAS1-F | ATGCCGCACCGTCATTGAG | qPCR |
| h-GAS1-R | TCATCGTAGTAGTCGTCCAGG | qPCR |
| h-CHRFAM7A-F | TGCATCTACCAGCATTTTCAGT | qPCR |
| h-CHRFAM7A-R | GCAATATCGCAGTTTGCAGCTAT | qPCR |
| h-EGFL7-F | TGAATGCAGTGCTAGGAGGG | qPCR |
| h-EGFL7-R | GCACACAGAGTGTACCGTCT | qPCR |
| h-LAMA4-F | CCACACTCGTCCTTCTCTCTC | qPCR |
| h-LAMA4-R | AGTTTCCGAACTGACCTAGCC | qPCR |

| | | |
|---|---|---|
| h-ICAM-F | ATGCCCAGACATCTGTGTCC | qPCR |
| h-ICAM-R | GGGGTCTCTATGCCCAACAA | qPCR |
| h-IGFBP3-F | AGACACACTGAATCACCTGAAGT | qPCR |
| h-IGFBP3-R | AGGGCGACACTGCTTTTTCTT | qPCR |
| h-PDGFRB-F | AGCACCTTCGTTCTGACCTG | qPCR |
| h-PDGFRB-R | TATTCTCCCGTGTCTAGCCCA | qPCR |
| h-GREM1-F | CGGAGCGCAAATACCTGAAG | qPCR |
| h-GREM1-R | GGTTGATGATGGTGCGACTGT | qPCR |
| h-F2RL1-F | CTGTGGGTCTTTCTTTTCCGAA | qPCR |
| h-F2RL1-R | CAAGGGGAACCAGATGACAGA | qPCR |
| h-TKTL1-F | ACAAGCAGTCAGATCCAGAGA | qPCR |
| h-TKTL1-R | TAGCTGGCCCTGTCGAAGTA | qPCR |
| h-PCK2-F | GCCATCATGCCGTAGCATC | qPCR |
| h-PCK2-R | AGCCTCAGTTCCATCACAGAT | qPCR |
| h-GALK1-F | TCGGTGGGCCAACTATGTC | qPCR |
| h-GALK1-R | GCAGCTATTGTGCCCGAGT | qPCR |
| h-PFKP-F | CGCCTACCTCAACGTGGTG | qPCR |
| h-PFKP-R | ACCTCCAGAACGAAGGTCCTC | qPCR |
| h-P16-F | ATGGAGCCTTCGGCTGACT | qPCR |
| h-P16-R | GTAACTATTCGGTGCGTTGGG | qPCR |
| h-P21-F | TGTCCGTCAGAACCCATGC | qPCR |
| h-P21-R | AAAGTCGAAGTTCCATCGCTC | qPCR |
| h-IL-6-F | ACTCACCTCTTCAGAACGAATTG | qPCR |
| h-IL-6-R | CCATCTTTGGAAGGTTCAGGTTG | qPCR |
| h-p53-F | CAGCACATGACGGAGGTTGT | qPCR |
| h-p53-R | TCATCCAAATACTCCACACGC | qPCR |
| h-TNFRSF9-F | AGCTGTTACAACATAGTAGCCAC | qPCR |
| h-TNFRSF9-R | GGACAGGGACTGCAAATCTGAT | qPCR |
| h-LBX1-F | CTCCAACAAGCCACTGACG | qPCR |
| h-LBX1-R | GCACAGCGAGTAACTTCTCCG | qPCR |
| h-ETS1-F | GATAGTTGTGATCGCCTCACC | qPCR |
| h-ETS1-R | GTCCTCTGAGTCGAAGCTGTC | qPCR |
| h-PAX3-F | AGCTCGGCGGTGTTTTTATCA | qPCR |
| h-PAX3-R | CTGCACAGGATCTTGGAGACG | qPCR |
| h-PLAU-F | GGGAATGGTCACTTTTACCGAG | qPCR |
| h-PLAU-R | GGGCATGGTACGTTTGCTG | qPCR |

| | | |
|---|---|---|
| h-CLU-F | CCAATCAGGGAAGTAAGTACGTC | qPCR |
| h-CLU-R | CTTGCGCTCTTCGTTTGTTTT | qPCR |
| h-S100A11-F | CTGAGCGGTGCATCGAGTC | qPCR |
| h-S100A11-R | TGTGAAGGCAGCTAGTTCTGTA | qPCR |
| h-EMP3-F | CCTGAATCTCTGGTACGACTGC | qPCR |
| h-EMP3-R | GCCATTCTCGCTGACATTACTG | qPCR |
| h-ANXA1-F | CTAAGCGAAACAATGCACAGC | qPCR |
| h-ANXA1-R | CCTCCTCAAGGTGACCTGTAA | qPCR |
| h-PLA2G4A-F | ATGGATGAAACTCTAGGGACAGC | qPCR |
| h-PLA2G4A-R | CTGGGCATGAGCAAACTTCAA | qPCR |
| h-SPARC-F | TGAGGTATCTGTGGGAGCTAATC | qPCR |
| h-SPARC-R | CCTTGCCGTGTTTGCAGTG | qPCR |
| h-FOSL2-F | CAGAAATTCCGGGTAGATATGCC | qPCR |
| h-FOSL2-R | GGTATGGGTTGGACATGGAGG | qPCR |
| h-CDKN1C-F | GCGGCGATCAAGAAGCTGT | qPCR |
| h-CDKN1C-R | GCTTGGCGAAGAAATCGGAGA | qPCR |
| h-CDKN1A-F | TGTCCGTCAGAACCCATGC | qPCR |
| h-CDKN1A-R | AAAGTCGAAGTTCCATCGCTC | qPCR |
| h-ATF3-F | CCTCTGCGCTGGAATCAGTC | qPCR |
| h-ATF3-R | TTCTTTCTCGTCGCCTCTTTTT | qPCR |
| h-GAPDH-F | TCGGAGTCAACGGATTTGGT | qPCR |
| h-GAPDH-R | TTGCCATGGGTGGAATCATA | qPCR |
| h-28S-F | CCCAGAAAAGGTGTTGGTTG | qPCR |
| h-28S-R | ATGGAACCCTTCTCCACTTC | qPCR |
| h-18S-F | GTAACCCGTTGAACCCCATT | qPCR |
| h-18S-R | CCATCCAATCGGTAGTAGCG | qPCR |
| h-5.8S-F | ACTCGGCTCGTGCGTC | qPCR |
| h-5.8S-R | GCGACGCTCAGACAGG | qPCR |
| h-5S-F | GTCTACGGCCATACCACCCTG | qPCR |
| h-5S-R | AAAGCCTACAGCACCCGGTAT | qPCR |
| h-CHIP-CBP-F1 | GGGTAAGCAAAGGGAAACAA | CHIP-qPCR |
| h-CHIP-CBP-R1 | AGTAGTGGGTGCAGTAGCGA | CHIP-qPCR |
| h-CHIP-CBP-F2 | CCTACACTCAGAAGCCCTCA | CHIP-qPCR |
| h-CHIP-CBP-R2 | TTGTTTCCCTTTGCTTACCC | CHIP-qPCR |
| h-CHIP-CBP-F3 | AGTGTCCAGCTATGCACTCG | CHIP-qPCR |
| h-CHIP-CBP-R3 | TTGCCTTGGTATGGCTCTAA | CHIP-qPCR |

| | | |
|---|---|---|
| h-CHIP-CBP-F4 | GAGCACTGGCCAGAAGAGA | CHIP-qPCR |
| h-CHIP-CBP-R4 | GCTTGCGTGTGAATATAGGC | CHIP-qPCR |
| h-CHIP-CBP-F5 | GCGCTTAGAGCCATACCAAG | CHIP-qPCR |
| h-CHIP-CBP-R5 | CCCTGTATTTGTGGAATGGG | CHIP-qPCR |
| h-CHIP-CBP-F6 | GCTTAGAGCCATACCAAGGC | CHIP-qPCR |
| h-CHIP-CBP-R6 | TTTCAAGGCACTTCTTCCCT | CHIP-qPCR |
| h-CHIP-CBP-F7 | ACAGGGAAGAAGTGCCTTGA | CHIP-qPCR |
| h-CHIP-CBP-R7 | GCCTGCTTTGACCTCCATAG | CHIP-qPCR |
| h-CHIP-CBP-F8 | GTCAAAGCAGGCCAGGTC | CHIP-qPCR |
| h-CHIP-CBP-R8 | GGGAGAGATTGGGAGATCAA | CHIP-qPCR |
| h-CHIP-PTEN-F | AGGATCTCTTCCCCAAGGTCTTTAGCCAAC | CHIP-PCR |
| h-CHIP-PTEN-R | AAGTGAGAAAAGACCTCCAGGGTGTGCCAA | CHIP-PCR |
| h-sh1-PAX7(S) | CGCGTGGAATCAAGTTCGGGAAGAATTCAAGAGATTCTTCCCGAACTT GATTCCTTTTTTGGAAAT | shRNA |
| h-sh1-PAX7(AS) | CGATTTCCAAAAAAGGAATCAAGTTCGGGAAGAATCTCTTGAATTCTTC CCGAACTTGATTCCA | shRNA |
| h-sh1-PAX7(S) | CGCGTGTCAGGTTTAGTGAGTTCGATTCAAGAGATCGAACTCACTAAA CCTGACTTTTTTGGAAAT | shRNA |
| h-sh1-PAX7(AS) | CGATTTCCAAAAAAGTCAGGTTTAGTGAGTTCGATCTCTTGAATCGAAC TCACTAAACCTGACA | shRNA |
| h-sh1-PTEN(S) | CGCGTGAGGTGAAGATATATTCCTCCAATTCAAGAGATTGGAGGAATAT ATCTTCACCTCTTTTTTGGAAAT | shRNA |
| h-sh1-PTEN(AS) | CGATTTCCAAAAAAGAGGTGAAGATATATTCCTCCAATCTCTTGAATTG GAGGAATATATCTTCACCTCA | shRNA |
| h-sh2-PTEN(S) | CGCGTGACTTGAAGGCGTATACAGGATTCAAGAGATCCTGTATACGCC TTCAAGTCTTTTTTGGAAAT | shRNA |
| h-sh2-PTEN(AS) | CGATTTCCAAAAAAGACTTGAAGGCGTATACAGGATCTCTTGAATCCTG TATACGCCTTCAAGTCA | shRNA |
| PAX7-promoter-F | CGGGGTACCGGATCTCTTCCCCAAGGTCTTTAGCCAACCTCA | Reporter |
| PAX7-promoter-R | CCGCTCGAGAGACTGCGGAGTGGATTTCGTTCTCGT | Reporter |
| h-Oct4-mF3 | ATTTGTTTTTTGGGTAGTTAAAGGT | Bisufite sequencing |
| h-Oct4-mR3 | CCAACTATCTTCATCTTAATAACATCC | Bisufite sequencing |
| CHIP-PAX7-F1 | AGAAGGAAGAGAAGTGGGAGAAGCACC | CHIP-PCR |
| CHIP-PAX7-R1 | CTGGGCAAGAACAACATCACTAAAGATACAACAAC | CHIP-PCR |
| CHIP-PAX7-F2 | GCTGAGCCAAGATCACACCACTGCACTTCAACCTG | CHIP-PCR |
| CHIP-PAX7-R2 | CTGCTCCCTCCCAAGACATAGGCCACAA | CHIP-PCR |
| CHIP-PAX7-F3 | TCAGGTTTGGGCAGATTATTTCAAGCTCACCCCTA | CHIP-PCR |
| CHIP-PAX7-R3 | TTGGTCCAGATGAGGTTGGCTAAAGACCTT | CHIP-PCR |

| | | |
|---|---|---|
| CHIP-PAX7-F4 | AGGATCTCTTCCCCAAGGTCTTTAGCCAAC | CHIP-PCR |
| CHIP-PAX7-R4 | AAGTGAGAAAAGACCTCCAGGGTGTGCCAA | CHIP-PCR |
| CHIP-PAX7-F5 | AGCTATGCACTCGCCCCACTCAGCTCAAGATGCTA | CHIP-PCR |
| CHIP-PAX7-R5 | CCCCTTGCAGCATGTGACAGATCTCCC | CHIP-PCR |
| CHIP-PAX7-F6 | CCCCTTCCAATTCGAAAGCTACTTGGCATCTGT | CHIP-PCR |
| CHIP-PAX7-R6 | CTTCTCCCTCGTCTTACACCCTCTGAAATTCCT | CHIP-PCR |
| CHIP-PAX7-F7 | GCAGACCCACTTTGCCAAGCCTCGGAAC | CHIP-PCR |
| CHIP-PAX7-R7 | CACCAGCTTTCACAGCCCCGTCCCCTA | CHIP-PCR |
| CHIP-PAX7-F8 | CCCCTCGCTTTTCCATTTCTCTTTCCCCAACCC | CHIP-PCR |
| CHIP-PAX7-R8 | CAGCCTCTCCAAGGACCCCGAGTTCCG | CHIP-PCR |
| CHIP-PAX7-F9 | GGTTCTGCCAGGCGCATCAGCCCGCACAA | CHIP-PCR |
| CHIP-PAX7-R9 | ACCCATGGTAGGTGGTTCGAAATAAAGGACT | CHIP-PCR |
| CHIP-PAX7-F10 | CCGGTCTTTACCCGCAGGGCTGAAATTCTGG | CHIP-PCR |
| CHIP-PAX7-R10 | CCTCCCTGCCTCCTCTCCCCTTCCTCACG | CHIP-PCR |
| CHIP-PAX7-F11 | GCACGGCAATGTCCAAACGAGAAGAAAGAGCTGAT | CHIP-PCR |
| CHIP-PAX7-R11 | CCCAGCGCACTAATCCGGCCTCGATCC | CHIP-PCR |
| CHIP-PAX7-F12 | CCCCCAATCTTCTGTGAGGAAATTGTGTAGACTT | CHIP-PCR |
| CHIP-PAX7-R12 | GATGGAGAGCAGACACTGAGTACACTCCC | CHIP-PCR |

## References


1. Iwamaru A*, et al.* A novel inhibitor of the STAT3 pathway induces apoptosis in malignant glioma cells both in vitro and in vivo. *Oncogene* **26**, 2435-2444 (2007).
2. Ferrajoli A*, et al.* WP1066 disrupts Janus kinase-2 and induces caspase-dependent apoptosis in acute myelogenous leukemia cells. *Cancer Res* **67**, 11291-11299 (2007).
3. Ramanathan RK*, et al.* A Phase I pharmacokinetic and pharmacodynamic study of PX-12, a novel inhibitor of thioredoxin-1, in patients with advanced solid tumors. *Clinical cancer research : an official journal of the American Association for Cancer Research* **13**, 2109-2114 (2007).
4. Myzak MC, Karplus PA, Chung FL, Dashwood RH. A novel mechanism of chemoprotection by sulforaphane: inhibition of histone deacetylase. *Cancer Res* **64**, 5767-5774 (2004).
5. Fahey JW*, et al.* Sulforaphane inhibits extracellular, intracellular, and antibiotic-resistant strains of Helicobacter pylori and prevents benzo[a]pyrene-induced stomach tumors. *Proc Natl Acad Sci U S A* **99**, 7610-7615 (2002).
6. Del Bufalo D*, et al.* Antiangiogenic potential of the Mammalian target of rapamycin inhibitor temsirolimus. *Cancer Res* **66**, 5549-5554 (2006).
7. Thomas GV*, et al.* Hypoxia-inducible factor determines sensitivity to inhibitors of mTOR in kidney cancer. *Nature medicine* **12**, 122-127 (2006).
8. Lefranc F*, et al.* Narciclasine, a plant growth modulator, activates Rho and stress fibers in glioblastoma cells. *Molecular cancer therapeutics* **8**, 1739-1750 (2009).
9. Ingrassia L*, et al.* Structure-activity relationship analysis of novel derivatives of narciclasine (an Amaryllidaceae isocarbostyril derivative) as potential anticancer agents. *Journal of medicinal chemistry* **52**, 1100-1114 (2009).
10. Zhang J, Hummersone M, Matthews CS, Stevens MF, Bradshaw TD. N3-Substituted Temozolomide Analogs Overcome Methylguanine-DNA Methyltransferase and Mismatch Repair Precipitating Apoptotic and Autophagic Cancer Cell Death. *Oncology* **88**, 28-48 (2014).
11. Rosenfeld MR*, et al.* A phase I/II trial of hydroxychloroquine in conjunction with radiation therapy and concurrent and adjuvant temozolomide in patients with newly diagnosed glioblastoma multiforme. *Autophagy* **10**, 1359-1368 (2014).
12. Hart MG, Grant R, Garside R, Rogers G, Somerville M, Stein K. Temozolomide for High Grade Glioma. *Cochrane Db Syst Rev*, (2008).
13. Lin Y, Kang T, Zhou BP. Doxorubicin enhances Snail/LSD1-mediated PTEN suppression in a PARP1-dependent manner. *Cell Cycle* **13**, 1708-1716 (2014).
14. Miyasaka A*, et al.* Anti-tumor activity of olaparib, a poly (ADP-ribose) polymerase (PARP) inhibitor, in cultured endometrial carcinoma cells. *BMC cancer* **14**, 179 (2014).
15. Josse R*, et al.* ATR Inhibitors VE-821 and VX-970 Sensitize Cancer Cells to Topoisomerase I Inhibitors by Disabling DNA Replication Initiation and Fork Elongation Responses. *Cancer Res* **74**, 6968-6979 (2014).
16. Platzer P*, et al.* Metabolism of camptothecin, a potent topoisomerase I inhibitor, in the isolated perfused rat liver. *Cancer chemotherapy and pharmacology* **45**, 50-54 (2000).
17. Kawasumi M*, et al.* Identification of ATR-Chk1 Pathway Inhibitors That Selectively Target p53-Deficient Cells without Directly Suppressing ATR Catalytic Activity. *Cancer Res*, (2014).
18. Ma H*, et al.* Effect and mechanism of Mitomycin C combined with recombinant adeno-associated virus type II against glioma. *International journal of molecular sciences* **15**, 1-14 (2014).
19. Kim R, Osaki A, Tanabe K, Kojima J, Toge T. A phase II trial of mitomycin C, 5 '-deoxy-5-fluorouridine, etoposide and medroxyprogesterone acetate (McVD-MPA) as a salvage chemotherapy to anthracycline-resistant tumor in relapsed breast cancer and its mechanism(s) of antitumor action. *Oncol Rep* **8**, 597-603 (2001).
